# The Feasibility and Test‐Retest Reliability of Wireless Dry‐Electrode EEG During a Dynamic Psychomotor Virtual Reality Task

**DOI:** 10.1002/brb3.70448

**Published:** 2025-04-27

**Authors:** Jazmin Morrone, Rik Mellor, Sidney Grosprêtre, Charles R. Pedlar, Giuseppe Cimadoro

**Affiliations:** ^1^ Faculty of Sport, Allied Health, and Performance Science St Mary's University London UK; ^2^ Laboratory Culture Sport Health Society (C3S‐UR 4660), Sport and Performance Department UFR STAPS, University of Franche‐Comté, 31 rue de l'Epitaphe Besançon France; ^3^ Institut Universitaire de France (IUF) Paris France; ^4^ Institute of Sport, Exercise and Health, Division of Surgery and Interventional Science University College London London UK

**Keywords:** alpha, combat sports, electroencephalogram, exergame, VR

## Abstract

**Purpose:**

Virtual reality (VR) offers immersive environments for studying psychomotor performance, but the reliability of dry‐electrode electroencephalography (EEG) in assessing brain activity during dynamic VR exergames (VRex) remains unclear. The present study investigated the feasibility and reliability of dry‐electrode EEG frequency band, with primary focus on alpha band activity.

**Methods:**

Ten amateur combat sports male participants (37 ± 11 years) volunteered for this study. The feasibility of dry‐electrode EEG recording during motion and test‐retest (24 h) reliability, was investigated. EEG measurements were obtained pre, post, and throughout a standardized boxing focus ball VRex session, comprising three 3‐min rounds interspersed with 1‐min rest intervals. EEG data were analyzed globally and at each electrode site, calculating average power spectral density values.

**Findings:**

ICCs data indicated poor‐to‐excellent (0.208–0.858) reliability across all measurements within the 4‐ to 30‐Hz frequency range. Poor‐to‐good reliability (0.393–0.636) was found across the task‐active VRex intervals. Electrode sites ranged in reliability from poor (electrode P3; 0.262) to excellent (electrode P4; 0.728), with higher reliability found in the alpha band across electrode sites compared to average spectral band values.

**Conclusion:**

The present study demonstrates the feasibility, although variable reliability, in neuronal detection during a dynamic VR task, using novel dry‐electrode EEG technology.

## Graphical abstract

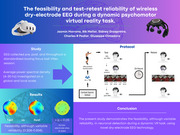

## 1 Introduction

Virtual reality (VR) encapsulates the simulation of the real‐world through interactive 3D modeling, often channeled via a headset with integrated screen to the user (Dwivedi et al. 2022). Simulated environments leveraging VR technology engage the human cognitive system through multisensory (e.g., visual, kinesthetic) motor function activation (Djebbara et al. 2019), therefore for its immersive aspects, offer applications within various fields. Resultantly, the implementation of VR has been found within the fields of education (e.g., Jensen and Konradsen 2018; Radianti et al. 2020; Snelson and Hsu 2020), healthcare (Guan et al. 2022; Kouijzer et al. 2023; Kumar Javvaji et al. 2024), and sports (Richlan et al. 2023; J. Wang 2012). For instance, VR exergames (VRex), defined as VR games requiring physical exertion from players (Yoo et al. 2017), are used to exercise psychomotor performance for real‐world training (Fahl et al. 2023). By integrating the cognitive efforts associated with motoric actions, VRex has been shown to alter cognitive functions and fine motor skills (Grosprêtre et al. 2023), as well as overall physiological response (Gomez et al. 2018), similar to the real‐world scenarios. Psychomotor performance, referring to the integration of cognitive and motor pathways, plays a critical role in both every day, and highly specific tasks, such as those found in sports (Morrone and Pedlar 2024a). For this reason, VR‐based training is of increasing interest (Richlan et al. 2023), especially within sports, where cognitive aspects such as high attention, alertness, visuospatial cognition, anticipatory skills, or reaction time are of critical importance (Morrone and Pedlar 2024b; Morrone and Minini 2023; Nuri et al. 2013; Russo and Ottoboni 2019).

Traditional training methods often lack the ability to isolate and target dynamic psychomotor performance in ecologically valid environments. As immersive and interactive environments allow the execution of more controlled and targeted scenarios involving psychomotor performance found within various daily tasks and sports skills (Yoo et al. 2017), VR acts as a means to enhance experimental control. For instance, VRex may be used to assess realistic human performance and the underlying psychomotor functions (e.g., motor coordination, reaction speed) in regulated yet simulated scenarios (Michalski et al. 2019). As such, VRex has been implemented within sports, such as golf (Harris et al. 2020), football (Fortes et al. 2021; Nambi et al. 2020), table tennis (Michalski et al. 2019), as well as combat sports, such as karate and boxing (Petri et al. 2019; Y. Wang et al. 2023). As VRex is used to support the enhancement of sports related psychomotor performance (Michalski et al. 2019), the neural adaptations associated with such tasks are of interest.

By further integrating VR technology with techniques, such as electroencephalogram (EEG), the quantification of the neurophysiological mechanisms associated with VR environment can be made (Choi et al. 2023; Ocklenburg and Peterburs 2023; Oliveira et al. 2018). The EEG is a non‐invasive and real‐time measure of neuroelectric activity (Biasiucci et al. 2019), which has been used to examine cognitive demands in sporting performance (Nakata et al. 2010; Park et al. 2015; C. H. Wang et al. 2019), providing insight on neural correlates linking cognitive skills and sport expertise (Cheron et al. 2016; Yarrow et al. 2009). However, traditional EEG instruments are limited by wired systems, sensitive to motion artifacts, and involve demanding operational requirements (e.g., long setup time), thereby constraining real‐world application across various environments such as clinical settings and scientific experiments (Zander et al. 2011). Traditional EEG during dynamic tasks has been used (e.g., in the MoBi‐EEG; Gramann et al. 2011), although these systems have predominantly been limited to walking movements, are high in cost, and complex to use. For this reason, cheaper and simpler (e.g., fast setup) instruments to measure brain electrical signals during a wider range of natural movements, including sports actions, is key to unlocking scientific advancements in the understanding of brain dynamics during movement.

Alternative advancements in EEG systems, such as wireless dry‐electrode EEG systems, offer potential to overcome such limitations (Hinrichs et al. 2020; C. H. Wang et al. 2019). For instance, advances in wireless dry‐electrode technology allow for application times of only a few minutes, offering an ease of use (i.e., by scientists and practitioners) and requiring less preparation and cleaning (Hinrichs et al. 2020; C. H. Wang et al. 2019). Such advancements therefore support the facilitation of rapid EEG data collection, creating new opportunities for EEG recording implementation (Drollinger et al. 2019; Rice et al. 2019). Although the integrity of detectable signal is maintained in dry‐electrode technology compared to traditional EEG systems (Hinrichs et al. 2020; Leach et al. 2020), the recording feasibility (i.e., signal integrity during motion) and the reliability of dry‐electrode EEG during dynamic VR tasks (e.g., VRex) is less known.

Interpretation of EEG power spectrum provides insight and understanding on the relationship between cortical areas and intrinsic features of task demand at hand (Oliveira et al. 2018). Notably, alpha EEG activity is directly linked with the accessing and processing of information which is involved in navigating the environment (e.g., sensorimotor processing, action observation, and movement planning; Klimesch 1997, 1999, 2012; Klimesch et al. 2011, 2007; Minarik et al. 2018; Quandt et al. 2011; ter Horst et al. 2013). This indicates that changes in alpha band activity acts as a means of evaluating the brain in response to multimodal sensory input from the environment (Klimesch 2012; Morrone and Minini 2023; Peylo et al. 2021). Further, the use of power frequency band investigation in adults shows adequate reliability across most bands and relational band measures (Lopez et al. 2023), with the highest reliability found centering the alpha band, compared to both lower (i.e., delta; 1–3 Hz) and higher (i.e., gamma; >30 Hz) frequency ranges (Hatz, Hardmeier, Bousleiman, et al. 2015; Höller, Uhl, et al., 2017). However, to the best of our knowledge, no reliability data are available for such EEG parameters when exposed to VRex. For this reason, alpha activity as a metric of EEG reliability acts as the primary form of evaluation within the present study.

The main aim of the present study was to investigate the feasibility and test‐retest reliability of neurophysiological indices collected with a dry‐electrode EEG headset, before, during, and after exposure to a dynamic psychomotor task, in the form of a boxing VRex. Both global and local evaluation is performed in terms of EEG‐based alpha band power spectrum activity, particularly due to the band's association with attentional and relevant cognitive demands. However, other frequency bands will be included in the evaluation, such as theta and beta frequency bands, due to their considered importance for cognitive and motor processing in athletes (Babiloni et al. 2008; Kao et al. 2013; Nakata et al. 2010). We hypothesized to find moderate‐to‐excellent reliability across the metrics analyzed, with the highest EEG recording reliability at resting conditions due to minimized motion. This is a consequence of the wireless dry‐electrode EEG headset's design to comply with the needs of scientific and clinical applications.

## 2 Materials and Methods

### 2.1 Participants

Ten healthy male participants (age = 37 ± 11 years; height = 178 ± 8 cm; body mass = 78 ± 10 kg) with an amateur combat sports background (i.e., familiar with fundamental boxing punching technique) volunteered for this study. A similar sample was used in previous EEG reliability research (Khanna et al. 2014). All were classified as novice to intermediate to capture brain activity across common amateur fighting skill ranges. All reported having normal or corrected vision (i.e., corrected vision was used if required), as well as being free from any neuromuscular pathological conditions. Ethical approval was granted by the St Mary's University Ethics Committee (SMU_ETHICS_2023‐24_494), in accordance with the ethical standards established in the Declaration of Helsinki. Informed written consent was provided by all participants.

### 2.2 Experimental Design

**FIGURE 1:**
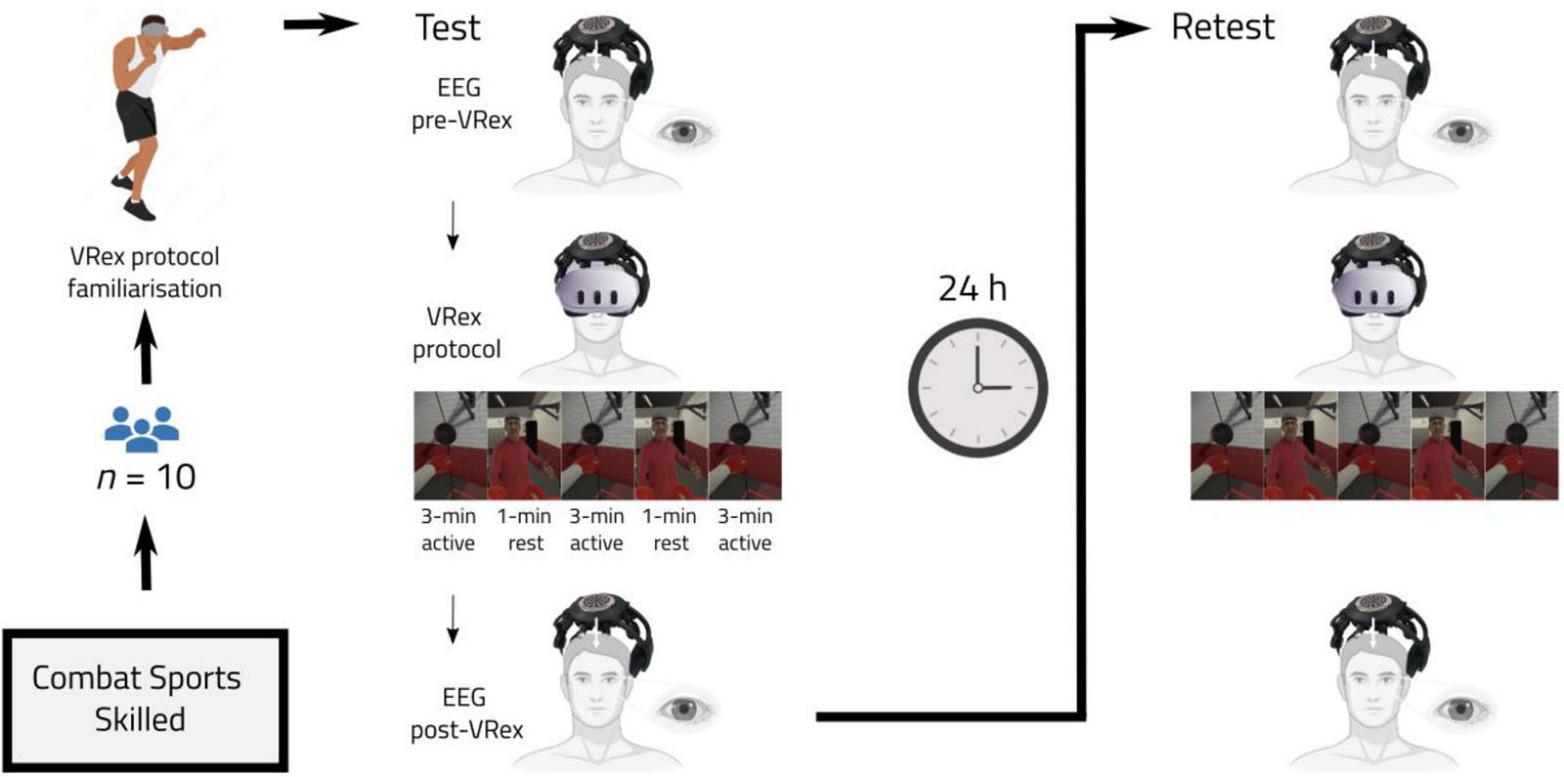
A schematic timeline of the experimental design (read from left, follow the arrows). The virtual reality headset and the EEG systems shown are the Meta Quest 3 (Meta Quest 3, Menlo Park, USA) and the wireless DSI‐7 system (Wearable Sensing, LLC, USA), respectively. EEG was recorded for 1 min before and after VRex exposure, and continuously recorded during the 11‐min VRex protocol.  VRex = virtual reality exergame; EEG = electroencephalogram.

A repeated measure design was used, whereby participants attended the lab twice, 24 h apart (test‐retest). The 24‐h interval was used to assess the short‐term stability of the dry‐electrode EEG frequency band evaluation. Each session involved a 5‐min minimal heart rate (HR) baseline to calculate HR reserve zones (Karvonen et al. 1957), a 1‐min pre‐VRex exposure EEG, an 11‐min task‐active VRex interval along with continuous EEG and HR recording, internal/external load quantification, and a 1‐min post‐VRex exposure EEG. Familiarization took place in the first session, which involved an introduction and demonstration of the equipment used, as well as a trial of the protocol. Participants repeated the exact protocol within the retest session (Figure 1). The independent variable was time, that is, test versus retest (and time within test/retest sessions). Dependent variables were the EEG metrics (as described below). HR peaks (beats per minute, bpm), punch count, punch maximal speed, and the perception of effort (CR100) were used to verify the standardization of the VRex execution. This entailed identical external load (i.e., punch count and speed) and comparable internal load (i.e., perception of effort and hear rate frequency) to ensure EEG recording occurred under the same conditions.

### 2.3 Procedures

At the start of each session, participants were fitted with the heart rate monitor and were instructed to lie supine on a portable massage plinth, relaxed with eyes closed. A silent 5‐min lying resting HR baseline was performed.

#### 2.3.1 EEG Setup and Protocol

**FIGURE 2:**
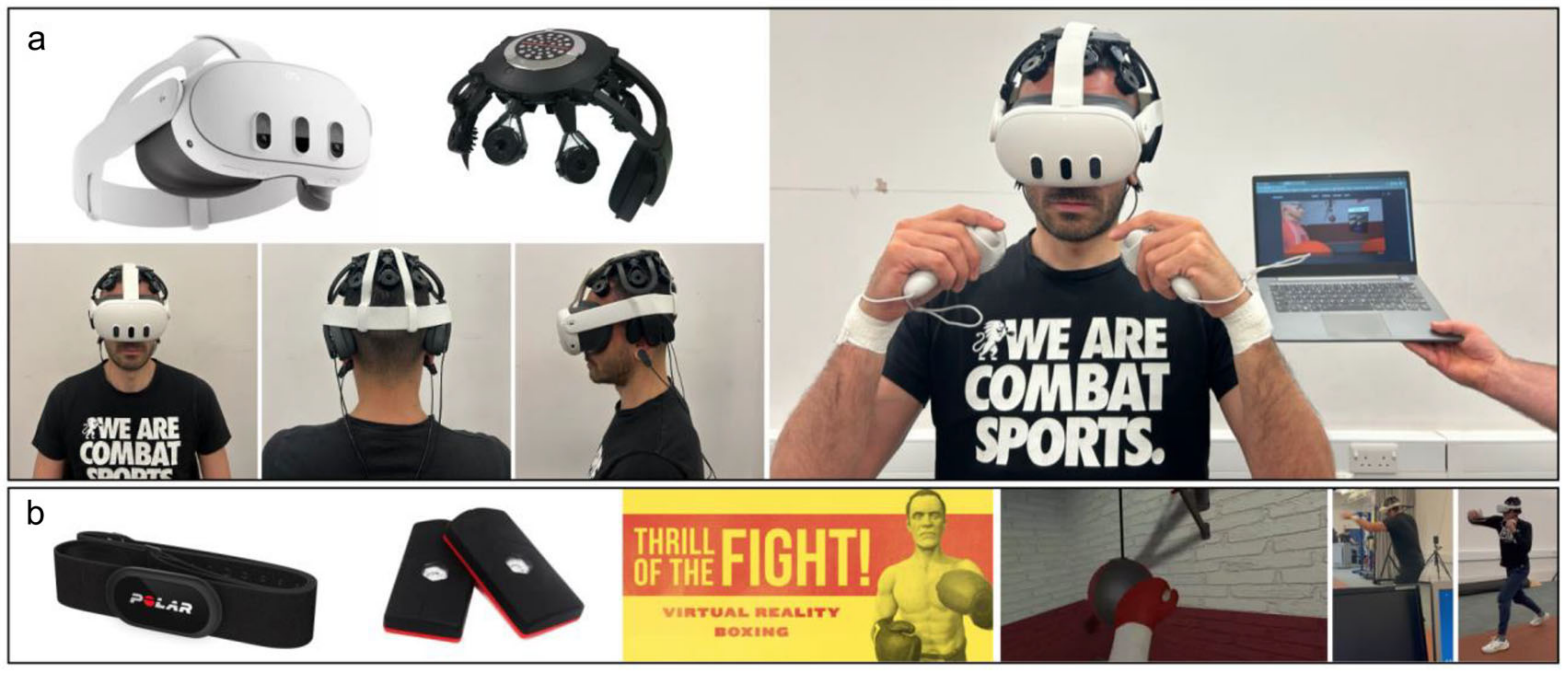
(a) Virtual reality (VR) headset (Meta Quest 3, Meta, USA), the dry‐electrode electroencephalogram (EEG) system (DSI‐7, Wearable Sensing, LLC, USA) with electrodes (F3, F4, C3, C4, Pz, P3, and P4) based upon the international 10–20 system, and view of the combined participant setup (front, back, side views) next to the casted VR boxing exergame. (b) From left: the heart rate hardware, the punch tracking device, the VR game, the VR task, example of participants during testing.

A validated wireless dry‐electrode 7‐sensor DSI‐7 EEG system (DSI‐7 SN:2449; Wearable Sensing, LLC, USA) was used (Kohli and Casson 2015; Mahdid et al. 2020; Snider et al. 2022). Recording occurred at a sampling rate of 300 Hz with the electrode setup (F3, F4, C3, C4, Pz, P3, P4) abiding by the 10–20 International System (as displayed in Figure 2, panel a) and was pre‐filtered by the DSI‐7 system with a high‐pass filter of 1 Hz, and a low‐pass filter of 50 Hz. The reference electrode was located on electrode site LE. Electrode impedance (<1 MΩ) was monitored both prior to and during all collections of data. To account for edge artifacts, buffer times of three seconds were used before and after each EEG collection (Delorme and Makeig 2004).

EEG was recorded for 1 min before and after VRex exposure, and continuously recorded during the 11‐min VRex protocol.  The 1‐min eyes open resting procedure performed both pre‐ and post‐VR exposure served as intraparticipant baselines (Cole et al., 2016; Olbrich et al. 2015; Popov et al. 2023; Raichle,^2015^). This was done to both confirm reliability at rest and evaluate the impact of the VRex protocol on subsequent resting conditions. Pre‐ and post‐VR exposure EEG measurements were collected within 5‐min prior and proceeding the task‐active interval, respectively. Both pre‐ and post‐VR exposure EEG collections were performed with participants seated in a visually controlled setting while staring at a blank wall with a marked single fixation point at eye level, with natural blinking rates.

#### 2.3.2 VRex Setup and Protocol

After the pre‐VR exposure EEG, participants were instructed to the VR setup. A VR headset (Meta Quest 3, Menlo Park, USA) with head‐mounted display (HMD; resolution of 2,064 × 2,208 pixels per eye; refresh rate of 120 Hz, 6 degrees of freedom, DoF) was employed (Figure 2, panel a). VR straps were adjusted to in accordance with (1) achieving a clear VR field of view, as confirmed by the participants; (2) a firmly secured (clear signal) EEG underneath the VR headset; and (3) each participants’ comfort. Participants were handed VR controllers then guided over to an open space into the VR boundary (3 × 3 m). The focus ball exergame within the “Thrill of The Fight” game (Sealost Interactive LLC, 2016) was used (Figure 2, panel b). The focus ball task was selected for its ability to stimulate attentional cues and complex reaction time (i.e., attention on a fast‐moving object) in controllable manner, which was expected to influence EEG metrics (Magosso et al. 2019; Ray and Cole 1985a, 1985b). Participants were guided to select the focus ball option via VR casting.

The VRex involved a 3‐ by 3‐min focus ball protocol (denoted as VR Round‐1, VR Round‐2, and VR Round‐3 for the three VR rounds) with a 1‐min rest between each subsequent round (Rest‐1 and Rest‐2). The protocol mimicked the typical boxing match format. The VRex was live‐casted to a laptop throughout the protocol to ensure that participants adhered to standardized instructions. To standardize the VRex, it was considered necessary that (1) repeated standardized boxing combinations were used to reasonably control for punching technique, which in turn created a fast random motion, thereby inducing necessary attentional cues and (2) punch quantity was identical for all participants to induce the same amount of focus ball collisions. A digital metronome sound (20 bpm) was used to dictate punching rhythm (i.e., one combo every 3 s) within every 3‐min round. Combos were sequentially: (1) three consecutive jabs, (2) cross + jab, and (3) cross + jab + cross. Straight punches were selected to avoid excessive levels of focus ball collision, for instance, as seen by those caused by hooks and/or uppercuts. Participants were also instructed to remain in a consistent field frame relative to the focus ball. If a combination was missed or performed incorrectly, the mistake was ignored, and participants were asked to continue with the next combination to minimize the risk of further mistakes. During the two 1‐min rest periods, participants remained standing, looking at the virtual coach presented in the virtual environment, to simulate real‐life training procedures and standardize cognitive demand at rest.

An initial familiarization was held, wherein participants were reminded of the protocol and were provided with a 1‐min trial bout to perform the protocol before data collection commenced. During the familiarization time, detailed and standardized instructions on the CR100 scale were also provided (see below; Pageaux et al. 2020, 2016). Once participants were comfortable with the protocol, data collection (EEG, HR monitor, and punch trackers) commenced, and the VR protocol began.

#### 2.3.3 VRex Standardization

To demonstrate the comparable effort across individuals during the 11‐min protocol (Figure 1), (i) HR was continuously monitored using an HR monitor (Polar P10; Kempele, Finland); (ii) wearable punch trackers (Hykso Inc., USA) were employed to reliably track punch count (Omcirk et al. 2021) and were strapped directly on top of the wrist as per manufacturer's instructions and previous research (Omcirk et al. 2021), using elasticated adhesive tape to secure and minimize movement (TigerTape; Physique Management Company Limited, UK); (iii) the perception of effort was collected immediately after each trial using the Borg CR100 (Borg and Borg 2002; Borg and Borg 2012; Hopkins 2000). Participants were asked to rate how hard overall it was for them to play the VRex game before participants completed the post‐VR 1‐min EEG. The CR100 scale ranges from 0 (no effort at all) to 100 (maximal effort); maximal effort was anchored as the greatest intensity of effort ever experienced by one participant during a physical task.

### 2.4 Data and Statistical Analysis

All data are presented as means ± standard deviation unless stated otherwise. All variables were tested for normality (Shapiro–Wilk test). When a variable was not normally distributed, a non‐parametric statical test was used. The alpha level for statistical significance was set at *α* ≤ 0.05. Partial eta squared (*η_p_* ^2^) are reported; thresholds for small, moderate, and large effects were set at 0.01, 0.07, and 0.14, respectively (Cohen 1988). Cohen's *d* were calculated when *t*‐tests were performed and thresholds for small, moderate, and large effects were set at 0.2, 0.5, and 0.8, respectively (Cohen 1988). For Wilcoxon rank tests, rank‐biserial correlation effect size thresholds were 0.10 small effect, 0.30 medium effect, and 0.50 large effect. Statistical analysis was performed using Jamovi version 2.5.6.0 (The Jamovi Project 2024) unless stated otherwise.

#### 2.4.1 EEG Analysis

All data were pre‐processed (see Appendix 1 for details), processed, and analyzed using MATLAB_R2021a (TheMathWorks, Inc., Natick, USA) and the EEGLAB 2024.0 package (Delorme and Makeig 2004). Topographical maps were achieved using EEGLAB (MATLAB Toolbox, USA). An initial group‐level global topographical map evaluation was performed using EEGLab's permutation statistical topographical software. Topographical plots (paired sample *t*‐tests) displayed the normalized interindividual differences (*p*‐values) in Power Spectrum Densities (µV^2^/Hz) between the test and the retest sessions across all electrode sites. This was performed within the frequency ranges of 4–30 Hz (i.e., frequencies ranging from the theta to the beta band) and 8–12 Hz (alpha band), independently. These plots were captured for the (1) pre‐VR exposure, (2) first VR round (VR Round‐1), (3) first 1‐min rest interval (Rest‐1), (4) second VR round (VR Round‐2), (5) second 1‐min rest interval (Rest‐2), (6) third VR round (VR Round‐3), and (7) post‐VR exposure, as an initial view of the main effects of the protocol.

Main effects of the sessions were initially tested using F‐tests in the form of repeated analyses of variance (ANOVAs) (Ip et al. 2018). Paired samples *t*‐tests were then performed to further evaluate the *p*‐values, effect size (*d*), and relevant descriptives (e.g., mean differences, MD). Such evaluation was performed for the global and local (i.e., at each electrode site) participant PSD averages, for all tasks individually (i.e., pre‐VR exposure, VR Round‐1, Rest‐1, VR Round‐2, Rest‐2, VR Round‐3, and post‐VR exposure). This was conducted both within each band frequency band, as well as overall across the 4–30 Hz frequency range. Averages across the specified frequency range were evaluated due to the reported variability in reliability found across individual frequency bands (e.g., Hatz, Hardmeier, Bousleiman, et al. 2015) and due to how averaging over a specified range supports higher reliability across EEG power band investigations (Höller, Uhl, et al., 2017). Local electrode investigation was performed across the specified frequency range, as well as for the alpha band independently, in alignment with the present work emphasis on this band.

#### 2.4.2 Heart Rate Analysis

Average absolute HR peaks and average relative (%) HR peaks were compared between the test and retest sessions using paired‐samples *t*‐tests. Holm–Bonferroni correction was applied to pairwise comparisons. Because HR was not synchronized, HR peaks were extracted using a 6‐s average, 3 s either side of the peak for respective VR‐boxing rounds, to have a good representation of the effort during boxing rounds. To assess if HR responses during the VR intervals were typical of intermittent exercise, Friedman's ANOVAs were used to analyze HR variations between rounds within each testing session separately (i.e., test and retest). Durbin–Conover pairwise analysis was used where significant differences occurred. Relative HR % was calculated using the following formula:

(1) (Peak6−sHR−RHR)/(HRmax−RHR)×100,

where RHR is resting HR average from 5‐min lying baselines; HRmax = 206.9 − (0.67 × age) (Jackson 2007).

#### 2.4.3 Punch Trackers

Average total punch count and average maximal punch speed across all rounds were compared using paired‐samples *t*‐tests. To confirm that punching output (i.e., external load) was the same round by round, total punch count and maximal punch speed (ms^−1^) by VRex round were extracted from the tracking application and assessed for differences between rounds using Friedman's tests for each session separately (i.e., test and retest).

#### 2.4.4 Perception of Effort

A paired‐samples *t*‐test was performed for the CPR100 scores to confirm that participants reported statistically identical perceptions at the end of the VR protocol.

#### 2.4.5 EEG Test‐Retest Reliability

For all parameters, the 24‐h inter‐day EEG relative reliability was assessed using the intraclass correlation coefficient (ICC; Ip et al. 2018; McGraw and Wong 1996; Popov et al. 2023). ICC consistency model (3, 1) was employed to compute the degree of reliability between the test and retest sessions of the specific raters involved in the study (Koo and Li 2016). The interpretation of ICC was followed: less than 0.40, poor reliability; between 0.40 and 0.59, fair reliability; between 0.60 and 0.74, good reliability; and between 0.75 and 1.00, excellent reliability (Cicchetti 1994; Tozzi et al. 2020). The absolute reliability was calculated with the typical error of measurement (i.e., standard error of measurement, SEM) to measure the noise of the parameters investigated (Hopkins 2000). Bland Altman's 95% limits of agreement were calculated to provide an additional representation of the agreement of the test‐retest results. As Bland–Altman plots portray the graphical agreement by using the statistical limits of agreement (i.e., based on the mean and standard deviation of the differences between two measurements), Bland–Altman plots were produced and represented accordingly.

## 3 Results

### 3.1 Verification of Protocol Standardization

#### 3.1.1 Heart Rate

Test data were normally distributed (*w* = 0.94, *p* = 0.092), and retest data were not normally distributed (*w* = 0.89, *p* = 0.004). The Wilcoxon rank test showed no significant difference in the three‐round‐median peak HR between Test (130.0 bpm ± 30.8 IQR) and Retest (132.5 bpm ± 26.0 IQR) sessions (*z* = 23, *p* = 0.695, *r* = −0.16 small). Repeated measure ANOVA revealed a simple effect of round on HR for the Test session (*f* = 14.93, *p* = 0.001, η^2^p = 0.62), and Friedman tests revealed significant differences for the Retest (*X*2 (2) = 14.6, *p* < 0.001) session. For the Test session, post hoc comparisons identified significant differences between R1 (132.3 bpm ± 24.9) and both (*p* = 0.018, *d* = 0.21 small) R2 (137.4 bpm ± 25.0) and R3 (143.7 bpm ± 28.3) (*p* = 0.007, *d* = 0.43 small), and between R2 and R3 (*p* = 0.007, *d* = 0.24 small). Pairwise Durbin–Conover comparisons within the Retest session identified significant differences between R1 (123.5 bpm ± 23.8 IQR) and both (*p* = 0.001, *d* = 0.53 moderate) R2 (130.0 bpm ± 26.5 IQR) and R3 (136.5 bpm ± 30.0 IQR) (*p* = 0.001, *d* = 0.83 large), and between R2 and R3 (*p* = 0.010, *d* = 0.26 small).

ANOVA showed a simple effect of round on relative HR % for both the Test and Retest (*f* = 38.80, *p* < 0.001, *η_p_* ^2^ = 0.76 large). Holm–Bonferroni corrected pairwise comparisons identified significant average differences between TestR1 (58.4 ± 5.69%) and TestR2 (64.8 ± 6.10%) (*t* = −5.75, *p* < 0.001), between TestR2 and TestR3 (69.8% ± 6.53) (*t* = −3.76, *p* = 0.004) and between TestR1 and TestR3 (*t* = −5.84, *p* < 0.001).

#### 3.1.2 Punch Trackers

Data were not normally distributed (*w* = 0.75, *p* = 0.003). The Wilcoxon rank test showed no significant difference between the median total punch count across rounds between Test (469.5 ± 44.0 IQR) and Retest (476.5 ± 26 IQR) (*z* = 13.5, *p* = 0.313, *r* = −0.40 medium). Friedman tests showed no significant difference in punch count between rounds for either Test (*X*2(2) = 5.74, *p* = 0.057) or Retest (*X*2(2) = 1.56, *p* = 0.459).

ANOVA showed no effect of round (F (2,18) = 2.350, *p* = 0.124), session (F (1,9) = 0.153, *p* = 0.705), or an interaction of round x session (F (2,18) = 0.770, *p* = 0.478) on average maximal punch speed (Test 11.9 ± 1.95 m/s and Retest 12.0 ± 2.0 m/s).

#### 3.1.3 Perception of Effort

The paired *t*‐test showed no significant difference in perception of effort recorded at the end of VRex (i.e., immediately after removing the headset) between Test (42.9 CR100 units ±18.9) and Retest (46 CR100 units ± 16.6) (t(9) = −1.46, *p* = 0.179, *d* = −0.461 moderate).

### 3.2 Global Inter‐Day Evaluation

#### 3.2.1 Global EEG 4–30 Hz

**FIGURE 3:**
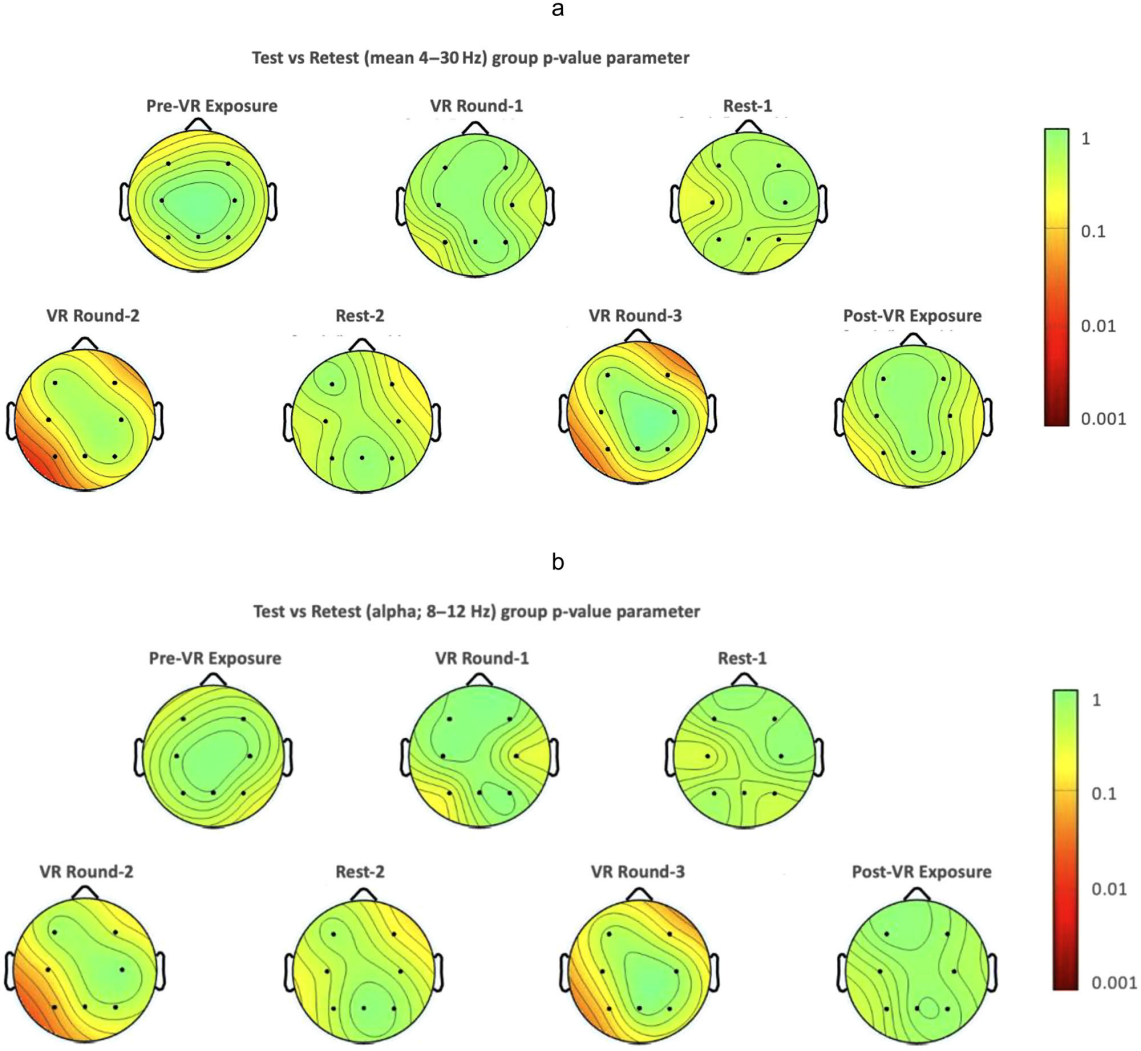
The statistical topographical plots display the normalized interindividual power spectrum densities (µV^2^/Hz) differences between the test and the retest sessions, with the local *p*‐value parameters represented. These topographical plots were computed for frequency range of 4–30 Hz (panel a; frequencies ranging from the theta to the beta band) and the alpha band (8–12 Hz; panel b) across all electrode sites. Plots were presented for the (i) pre‐virtual reality (VR) exposure, (ii) VR Round‐1, (iii) the first rest interval (Rest‐1), (iv) VR Round‐2, (v) the second rest interval (Rest‐2), (vi) VR Round‐3, and (vii) post‐VR exposure. Such topographical maps demonstrate that across electrode sites, no significant differences were found when comparing test and retest sessions, for neither the pre‐, during, nor the post‐VR EEG collections. This was displayed both within the alpha band, as well as across the theta–beta band frequency range.

**FIGURE 4:**
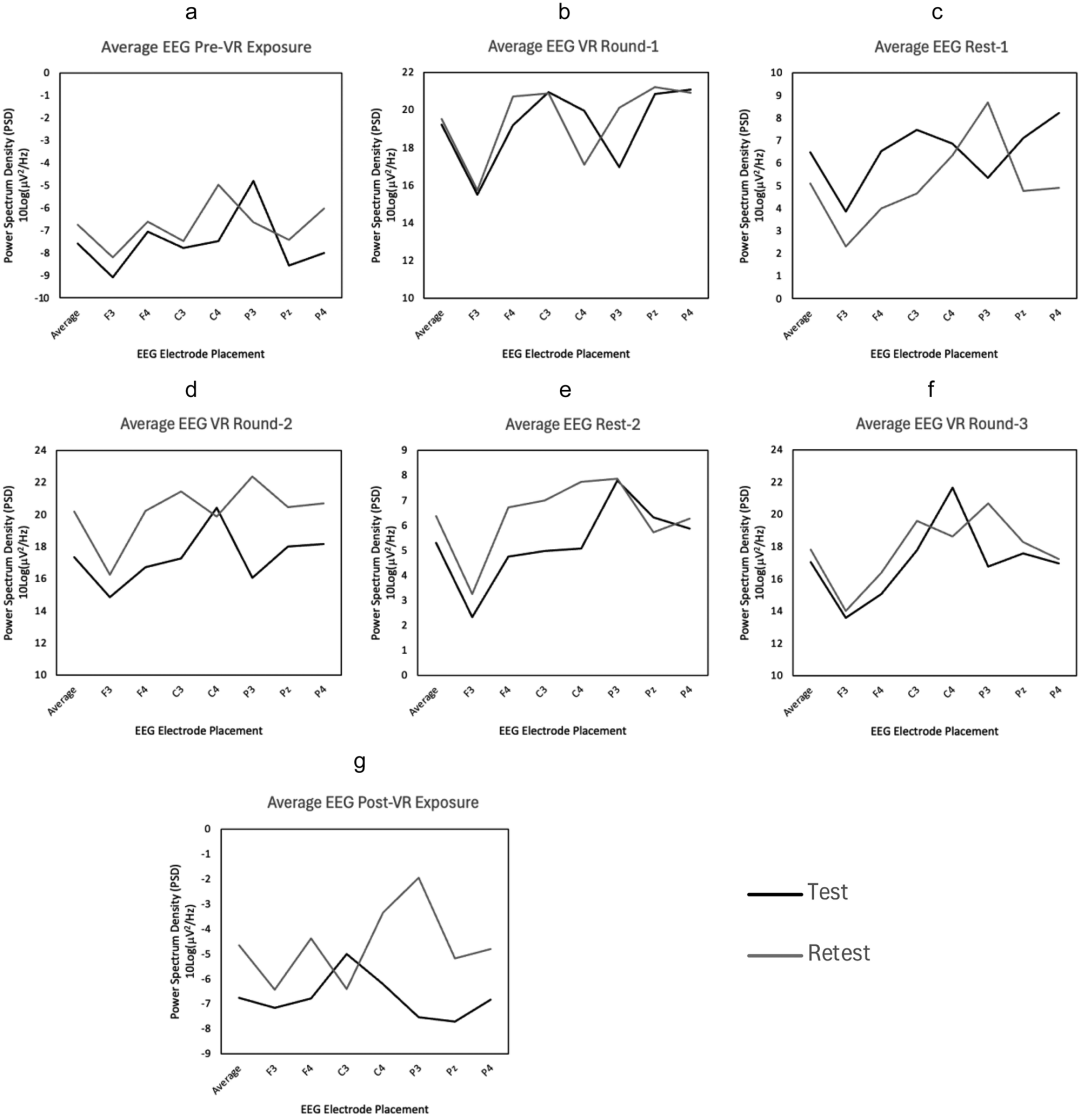
Mean EEG spectral power estimate comparisons between conditions for all electrode placements for all participants for the average (4–30 Hz) frequency range. These are presented for the (a) pre‐virtual reality (VR) exposure, (b) VR Round‐1, (c) the first rest interval (Rest‐1), (d) VR Round‐2, (e) the second rest interval (Rest‐2), (f) VR Round‐3, and (g) post‐VR exposure.

The normalized permutational statistics topographical plots displayed no statistical differences across electrode sites across all tasks for the overall frequency range of 4–30 Hz (see Figure 3). F‐tests and *t*‐tests revealed no significant differences between global EEG metrics across all tasks for the test‐retest sessions (*p* > 0.05; see Table 1). ICC reports of the test‐retest evaluation of the global power spectrum EEG data ranged from poor (i.e., second rest interval Rest‐2; ICC = 0.208, CI = [−0.352, 0.658]) to excellent reliability (for the pre‐VR 1‐min EEG; ICC = 0.858, CI = [0.608, 0.953]) within the average frequency band range. Inter‐day *t*‐test and reliability and agreement (SEM) results for continuous EEG are presented in Table 1. See Figure 4 for mean EEG spectral power estimate comparisons between conditions.

**TABLE 1:** Global frequency band evaluation (4–30 Hz).

EEG power spectrum	Construct	Condition	Test (day 1) (24 h)	Retest (day 2) (24 h)	MD	Paired *t*‐test *p*	Effect size *d*	ICC (3,1) consistency [CIs 95%]	SEM
			Mean ±SD (10log(µV^2/Hz^))						
All (4–30 Hz)		Pre‐VR Exposure	−7.60 ± 3.88	−6.76 ± 5.24	−0.836	0.310	−0.340	**0.858 [0.608 0.953**]	1.738
		VR Round‐1	19.2 ± 9.19	19.5 ± 7.39	−0.315	0.907	−0.038	0.542 [0.029 0.829]	5.574
		Rest‐1	6.49 ± 11.78	5.10 ± 9.74	1.390	0.702	0.125	0.506 [−0.020 0.813]	7.512
		VR Round‐2	17.3 ± 7.61	20.2 ± 8.66	−2.840	0.344	−0.316	0.393 [−0.162 0.759]	6.354
		Rest‐2	5.30 ± 8.53	6.37 ± 10.07	−1.070	0.786	−0.088	0.208 [−0.352 0.658]	8.143
		VR Round‐3	17.1 ± 7.68	17.8 ± 7.75	−0.777	0.742	−0.107	0.591 [0.100 0.850]	4.888
		Post‐VR Exposure	−6.75 ± 4.45	−4.65 ± 7.65	−2.100	0.293	−0.353	0.575 [0.076 0.843]	4.201
Theta (4–7 Hz)	Drowsiness, meditation, daydreaming, memory encoding, and retrieval	Pre‐VR Exposure	−8.35 ± 4.28	−6.45 ± 6.29	−1.900	0.092	−0.597	**0.824 [0.532 0.941]**	2.253
		VR Round‐1	19.9 ± 9.47	20.1 ± 7.65	−0.157	0.957	−0.018	0.495 [−0.036 0.808]	6.032
		Rest‐1	9.51 ± 13.5	6.52 ± 10.3	2.990	0.463	0.242	0.489 [−0.043 0.805]	8.546
		VR Round‐2	19.2 ± 8.67	21.0 ± 9.39	−1.810	0.571	−0.186	0.450 [−0.093 0.787]	6.637
		Rest‐2	8.17 ± 10.43	7.68 ± 9.47	0.488	0.896	0.043	0.384 [−0.172 0.754]	7.694
		VR Round‐3	18.6 ± 8.80	18.2 ± 7.76	0.476	0.859	0.058	0.540 [0.027 0.828]	5.557
		Post‐VR Exposure	−7.47 ± 5.07	−4.83 ± 8.71	−2.64	0.206	−0.431	**0.630 [0.162 0.867]**	4.334
Alpha (8–12 Hz)	Attention, mental calmness, mind wandering, creativity	Pre‐VR Exposure	−7.67 ± 4.23	−6.83 ± 5.87	−0.839	0.372	−0.297	**0.850 [0.590 0.950]**	1.983
		VR Round‐1	20.0 ± 9.16	20.5 ± 7.31	−0.444	0.873	−0.052	0.513 [−0.012 0.816]	5.571
		Rest‐1	7.72 ± 12.02	5.68 ± 9.71	2.030	0.579	0.182	0.506 [−0.021 0.813]	7.619
		VR Round‐2	17.1 ± 7.62	21.0 ± 8.75	−2.490	0.409	−0.274	**0.653 [−0.158 0.760]**	6.353
		Rest‐2	6.65 ± 8.55	7.22 ± 9.86	−0.567	0.886	−0.047	0.186 [−0.372 0.645]	8.149
		VR Round‐3	17.8 ± 7.69	18.6 ± 7.68	−0.787	0.737	−0.110	0.595 [0.107 0.852]	4.844
		Post‐VR Exposure	−7.74 ± 5.07	−4.83 ± 8.71	−2.640	0.206	−0.431	**0.630 [0.162 0.867]**	4.333
Low beta (13–16 Hz)	Focused attention, sensory processing, motor preparation	Pre‐VR Exposure	−6.86 ± 3.82	−6.63 ± 5.26	−0.235	0.777	−0.092	**0.859 [0.612 0.954]**	1.717
		VR Round‐1	19.8 ± 9.14	20.3 ± 7.39	−0.485	0.852	−0.061	0.573 [0.073 0.843]	5.373
		Rest‐1	5.61 ± 11.4	6.52 ± 10.3	−0.902	0.800	−0.083	0.532 [0.015 0.825]	7.361
		VR Round‐2	17.6 ± 7.46	20.7 ± 8.38	−3.090	0.301	−0.347	0.372 [−0.285 0.748]	6.288
		Rest‐2	4.27 ± 7.59	6.09 ± 10.72	−1.820	0.666	−0.141	0.076 [−0.464 0.574]	8.754
		VR Round‐3	17.4 ± 7.39	18.6 ± 7.92	−1.190	0.611	−0.167	0.593 [0.104 0.851]	4.848
		Post‐VR Exposure	−5.25 ± 4.04	−3.39 ± 7.54	−1.760	0.389	−0.826	0.490 [−0.042 0.806]	4.309
High beta (17–30 Hz)	Active information processing, alertness	Pre‐VR Exposure	−7.50 ± 3.63	−7.14 ± 4.15	−0.839	0.541	−0.201	**0.896 [0.707 0.966]**	1.256
		VR Round‐1	17.1 ± 10.34	17.3 ± 7.81	−0.173	0.950	−0.020	0.605 [0.122 0.856]	5.700
		Rest‐1	3.12 ± 10.76	3.14 ± 8.97	−0.022	0.995	−0.002	0.495 [−0.033 0.809]	6.933
		VR Round‐2	14.2 ± 7.63	18.1 ± 8.39	−3.970	0.191	−0.447	0.388 [−0.167 0.756]	6.275
		Rest‐2	2.12 ± 7.80	4.49 ± 10.40	−2.370	0.559	−0.192	0.129 [−0.421 0.609]	8.455
		VR Round‐3	14.4 ± 7.93	16.0 ± 7.99	−1.610	0.487	−0.229	**0.624 [0.152 0.864]**	4.856
		Post‐VR Exposure	−6.45 ± 3.54	−5.52 ± 6.87	−0.936	0.603	−0.170	0.522 [0.001 0.820]	3.744

*Note*: Relative reliability (intraclass correlation coefficient; ICC), agreement (standard error of measurement; SEM), and mean difference (MD) of the average (4–30 Hz) and individual EEG power spectrum bands were decomposed into the following: theta; 4–7 Hz, alpha; 8–12 Hz, low beta; 13–16 Hz, high beta; 17–30 Hz. EEG metrics were recorded for pre‐virtual reality (VR) exposure, during three rounds of VR (denoted as VR Round‐1, VR Round‐2, and VR Round‐3, respectively) during two intermediate rest intervals between each VR round (denoted as Rest‐1 and Rest‐2, respectively) and during a post‐VR exposure condition. CIs = 95% confidence intervals; SD = standard deviation; *n* = 10. ICC ≥ 0.6 (i.e., good and excellent) are represented in bold. EEG power band constructs were adapted from Schomer and Lopes da Silva (2010).

#### 3.2.2 Alpha Band EEG

The normalized permutational statistics topographical plots displayed no statistical differences across electrode sites across all tasks for the alpha frequency range (see Figure 3). F‐tests and *t*‐tests revealed no significant differences between global EEG metrics across all tasks for the test‐retest sessions (*p* > 0.05; see Table 1). ICC reports of the test‐retest evaluation of the global power spectrum EEG data ranged from poor (i.e., second rest interval Rest‐2; ICC = 0.186, CI = [−0.372, 0.645]) to excellent reliability (for the pre‐VR exposure EEG; ICC = 0.850, CI = [0.590, 0.950]) within the alpha frequency band range. Inter‐day *t*‐test and reliability and agreement (SEM) results for continuous EEG are presented in Table 1.

#### 3.2.3 Theta Band EEG

*t*‐Tests revealed no significant differences between global EEG metrics across all tasks for the test‐retest sessions (*p* > 0.05; see Table 1). ICC reports of the test‐retest evaluation of the global power spectrum EEG data ranged from poor (i.e., second rest interval Rest‐2; ICC = 0.384, CI = [−0.172, 0.754]) to excellent reliability (for the pre‐VR exposure EEG; ICC = 0.824, CI = [0.532, 0.941]) within the theta frequency band range. Inter‐day *t*‐test and reliability and agreement (SEM) results for continuous EEG are presented in Table 1.

#### 3.2.4 Low‐Beta Band EEG

*t*‐Tests revealed no significant differences between global EEG metrics across all tasks for the test‐retest sessions (*p* > 0.05; see Table 1). ICC reports of the test‐retest evaluation of the global power spectrum EEG data ranged from poor (i.e., second rest interval Rest‐2; ICC = 0.0.076, CI = [−0.464, 0.574]) to excellent reliability (for the pre‐VR exposure EEG; ICC = 0.859, CI = [0.612, 0.954]) within the low‐beta frequency band range. Inter‐day *t*‐test and reliability and agreement (SEM) results for continuous EEG are presented in Table 1.

#### 3.2.5 High‐Beta Band EEG

*t*‐Tests revealed no significant differences between global EEG metrics across all tasks for the test‐retest sessions (*p* > 0.05; see Table 1). ICC reports of the test‐retest evaluation of the global power spectrum EEG data ranged from poor (i.e., second rest interval Rest‐2; ICC = 0.129, CI = [−0.421, 0.7609]) to excellent reliability (for the pre‐VR exposure EEG; ICC = 0.895, CI = [0.707, 0.966]) within the high‐beta frequency band range. Inter‐day *t*‐test and reliability and agreement (SEM) results for continuous EEG are presented in Table 1.

### 3.3 Inter‐Day Electrode Evaluation

#### 3.3.1 Average Pre‐ and Post‐VR Exposure EEG

No main effects of the sessions on the global EEG results were reported for the pre‐VR exposure EEG (*F*(1,10) = 1.160, *p* = 0.310, *η* ^2^ = 0.009), as well as for any electrode placements (*p* > 0.05; see Table 1). Global EEG metrics over the specified frequency bands showed excellent test‐retest reliability (0.858; CI = [0.607, 0.953]) for the pre‐VR exposure EEG. When investigating individual electrode sites, excellent reliability was displayed in electrode placement F4 (0.777; CI = [0.431, 0.924]), good reliability was found in electrode sites F3, C3, C4, and P4 (0.678‐0.707), with poor‐to‐fair reliability found in electrodes Pz and P3 with ICC values at 0.216 and 0.498, respectively. Agreement (SEM) was found in Tables 1 and 2.

**TABLE 2:** Averages across all specified bands (i.e., theta to high beta; 4–30 Hz).

EEG (4–30 Hz)		Test (day 1) (2 h)	Retest (day 2) (2 h)	MD	Paired *t*‐test *p*	Effect size *d*	ICC (3,1) Consistency [CIs 95%]	SEM
		Mean ± SD (10Log(µV^2/Hz^))						
Global	Pre‐VR Exposure	−7.60 ± 3.88	−6.76 ± 5.24	−0.836	0.310	−0.340	**0.858 [0.608 0.953]**	1.738
	VR Round‐1	19.2 ± 9.19	19.5 ± 7.39	−0.315	0.907	−0.038	0.542 [0.029 0.829]	5.574
	Rest‐1	6.49 ± 11.78	5.10 ± 9.74	1.390	0.702	0.125	0.506 [−0.020 0.813]	7.512
	VR Round‐2	17.3 ± 7.61	20.2 ± 8.66	−2.840	0.344	−0.316	0.393 [−0.162 0.759]	6.354
	Rest‐2	5.30 ± 8.53	6.37 ± 10.07	−1.070	0.786	−0.088	0.208 [−0.352 0.658]	8.143
	VR Round‐3	17.1 ± 7.68	17.8 ± 7.75	−0.777	0.742	−0.107	0.591 [0.100 0.850]	4.888
	Post‐VR Exposure	−6.75 ± 4.45	−4.65 ± 7.65	−2.100	0.293	−0.353	0.575 [0.076 0.843]	4.201
F3	Pre‐VR Exposure	−9.09 ± 4.02	−8.19 ± 4.22	−0.893	0.398	−0.280	**0.707 [0.294 0.897]**	2.228
	VR Round‐1	15.5 ± 9.80	15.7 ± 6.83	−0.220	0.938	−0.025	0.504 [−0.023 0.812]	5.871
	Rest‐1	3.86 ± 11.40	2.31 ± 8.58	1.550	0.661	0.143	0.462 [−0.078 0.793]	7.316
	VR Round‐2	14.9 ± 10.57	16.2 ± 7.71	−1.380	0.647	−0.150	0.532 [0.014 0.824]	6.271
	Rest‐2	2.33 ± 8.54	3.25 ± 9.20	−0.919	0.807	−0.079	0.197 [−0.362 0.651]	7.792
	VR Round‐3	13.6 ± 7.72	14.0 ± 7.72	−0.432	0.854	−0.060	0.520 [−0.002 0.819]	4.838
	Post‐VR Exposure	−7.15 ± 3.32	−6.44 ± 7.29	−0.712	0.739	−0.113	0.417 [−0.134 0.771]	4.266
F4	Pre‐VR Exposure	−7.06 ± 5.46	−6.61 ± 4.48	−0.449	0.691	−0.310	**0.777 [0.431 0.924]**	2.346
	VR Round‐1	19.2 ± 9.43	20.7 ± 6.77	−1.530	0.640	−0.153	0.296 [−0.267 0.708]	6.789
	Rest‐1	6.53 ± 11.87	4.00 ± 8.22	2.530	0.540	0.201	0.270 [−0.292 0.694]	8.612
	VR Round‐2	16.7 ± 7.23	20.2 ± 7.71	−3.500	0.238	−0.400	0.312 [−0.250 0.717]	6.197
	Rest‐2	4.74 ± 7.87	6.72 ± 7.30	−1.980	0.572	−0.186	0.045 [−0.488 0.554]	7.298
	VR Round‐3	15.0 ± 7.28	16.4 ± 6.98	−1.340	0.504	−0.220	**0.653 [0.199 0.876]**	4.182
	Post‐VR Exposure	−6.78 ± 4.23	−4.38 ± 8.96	−2.400	0.259	−0.381	0.596 [0.108 0.852]	4.453
C3	Pre‐VR Exposure	−7.78 ± 5.48	−7.47 ± 5.66	−0.306	0.838	−0.067	**0.687 [0.258 0.890]**	3.092
	VR Round‐1	21.0 ± 10.35	20.9 ± 9.63	0.059	0.986	0.006	0.480 [−0.055 0.801]	7.103
	Rest‐1	7.48 ± 11.1	4.65 ± 11.2	2.830	0.435	0.258	0.527 [0.007 0.822]	7.619
	VR Round‐2	17.2 ± 7.18	21.4 ± 11.10	−4.200	0.204	−0.433	0.459 [−0.082 0.791]	6.872
	Rest‐2	4.98 ± 9.14	6.99 ± 12.54	−2.010	0.691	−0.130	0.046 [−0.487 0.554]	10.493
	VR Round‐3	17.8 ± 8.75	19.6 ± 10.35	−1.800	0.513	−0.215	**0.634 [0.168 0.868]**	5.767
	Post‐VR Exposure	−5.01 ± 9.00	−6.41 ± 8.33	1.400	0.548	0.197	**0.683 [0.250 0.940]**	4.861
C4	Pre‐VR Exposure	−7.91 ± 4.07	−8.93 ± 6.60	−2.940	0.061	−0.675	**0.685 [0.255 0.889]**	3.076
	VR Round‐1	20.0 ± 8.53	17.1 ± 7.28	2.850	0.258	0.382	0.558 [0.051 0.836]	5.274
	Rest‐1	6.87 ± 12.9	6.35 ± 10.8	0.512	0.887	0.046	**0.605 [0.122 0.856]**	7.417
	VR Round‐2	20.4 ± 8.07	19.9 ± 9.42	0.544	0.858	0.058	0.470 [−0.068 0.796]	6.297
	Rest‐2	5.07 ± 11.1	7.74 ± 11.7	−2.670	0.491	−0.227	0.484 [−0.050 0.803]	8.114
	VR Round‐3	21.7 ± 10.99	18.6 ± 8.60	3.010	0.399	0.280	0.415 [−0.136 0.770]	7.523
	Post‐VR Exposure	−6.21 ± 4.72	−3.36 ± 8.87	−2.850	0.278	−0.365	0.396 [−0.159 0.760]	5.526
P3	Pre‐VR Exposure	−4.81 ± 4.99	−6.63 ± 7.46	1.820	0.393	0.284	0.498 [−0.031 0.809]	4.486
	VR Round‐1	17.0 ± 8.53	20.1 ± 8.25	−3.140	0.291	−0.355	0.446 [−0.098 0.785]	6.244
	Rest‐1	5.35 ± 13.6	8.69 ± 13.0	−3.340	0.530	−0.207	0.292 [−0.271 0.706]	11.108
	VR Round‐2	16.1 ± 8.84	22.4 ± 10.35	−6.310	0.122	−0.540	0.262 [−0.301 0.689]	8.267
	Rest‐2	7.79 ± 10.9	7.88 ± 13.1	−0.089	0.985	−0.006	0.337 [−0.224 0.730]	9.657
	VR Round‐3	16.8 ± 8.52	20.7 ± 9.96	−3.880	0.219	−0.418	0.499 [−0.031 0.810]	6.564
	Post‐VR Exposure	−7.54 ± 4.53	−1.95 ± 9.80	−5.600	0.047*	−0.728	0.492 [−0.039 0.807]	5.437
Pz	Pre‐VR Exposure	−8.55 ± 7.68	−7.42 ± 7.50	−1.130	0.723	−0.116	0.216 [−0.344 0.663]	6.958
	VR Round‐1	20.8 ± 7.80	21.2 ± 10.75	−0.379	0.904	−0.039	0.507 [−0.020 0.813]	6.509
	Rest‐1	7.13 ± 10.7	4.78 ± 11.2	2.350	0.429	0.262	**0.674 [0.235 0.884]**	6.249
	VR Round‐2	18.0 ± 5.95	20.5 ± 10.39	−2.450	0.472	−0.237	0.276 [−0.287 0.697]	7.147
	Rest‐2	6.33 ± 8.42	5.73 ± 11.54	0.595	0.881	0.049	0.316 [−0.246 0.719]	8.199
	VR Round‐3	17.6 ± 7.31	18.3 ± 8.96	−0.715	0.807	−0.078	0.436 [−0.110 0.780]	6.048
	Post‐VR Exposure	−7.71 ± 4.82	−5.19 ± 8.72	−2.520	0.244	−0.394	0.588 [0.096 0.849]	4.523
P4	Pre‐VR Exposure	−7.99 ± 3.72	−6.04 ± 6.88	−1.950	0.197	−0.440	**0.678 [0.242 0.886]**	3.139
	VR Round‐1	21.1 ± 12.84	20.9 ± 8.57	0.154	0.968	0.013	0.464 [−0.075 0.794]	7.875
	Rest‐1	8.22 ± 14.37	4.92 ± 8.93	3.300	0.449	0.250	0.408 [−0.144 0.766]	9.152
	VR Round‐2	18.1 ± 10.08	20.7 ± 9.59	−2.560	0.503	−0.220	0.324 [−0.237 0.723]	8.013
	Rest‐2	5.88 ± 8.74	6.28 ± 9.27	−0.396	0.927	−0.030	0.000 [−0.521 0.521]	8.770
	VR Round‐3	16.9 ± 7.74	17.2 ± 8.78	−0.283	0.897	−0.042	**0.697 [0.276 0.894]**	4.517
	Post‐VR Exposure	−6.83 ± 3.99	−4.81 ± 7.06	−2.020	0.259	−0.381	0.573 [0.073 0.843]	3.748

*Note*: Reliability (intraclass correlation coefficient; ICC), agreement (standard error of measurement; SEM), and mean difference (MD) for the average global (i.e., across all electrodes) and local (i.e., at each electrode site; F3, F4, C3, C4, P3, Pz, and P4) across the EEG power spectrum (4–30 Hz). EEG metrics were recorded for pre‐virtual reality (VR) exposure during three rounds of VR (denoted as VR Round‐1, VR Round‐2, and VR Round‐3, respectively) during two intermediate rest intervals between each VR round (denoted as Rest‐1 and Rest‐2, respectively), and during a post‐VR exposure condition. CIs = 95% confidence intervals; SD = standard deviation; *n* = 10. ICC ≥ 0.6 (i.e., good and excellent) are represented in bold.

*p < 0.05.

Similarly, neither main effects were reported on the global EEG results for the post‐VR exposure EEG (*F*(1,10) = 1.250, *p* = 0.293, *η* ^2^ = 0.029), nor for all electrode placements (*p* > 0.05; see Table 1), apart from electrode P3 (*p* = 0.047). Global EEG metrics showed fair test‐retest reliability (0.575; CI = [0.076, 0.843]) for the post‐VR exposure EEG. When investigating individual electrode sites, good reliability was displayed in electrode placement C3 (0.683; CI = [0.250, 0.888]) and fair reliability was found in electrode sites F3, F4, P3, Pz, and P4 (0.417–0.596), with poor reliability found in electrodes C4 (0.395; CI = [−0.159, 0.760]). Agreement (SEM) was found in Tables 1 and 2.

#### 3.3.2 Alpha Pre‐ and Post‐VR Exposure EEG

No main effects of the sessions on the global EEG results were reported for the pre‐VR exposure EEG (*F*(1,10) = 0.884, *p* = 0.372, *η* ^2^ = 0.007), as well as for any electrode placements (*p* > 0.05; see Table 1). Global EEG metrics over the alpha frequency bands showed excellent test‐retest reliability (0.850; CI = [0.590, 0.950]) for the pre‐VR exposure EEG. When investigating individual electrode sites, excellent reliability was displayed in electrode placement F4 (0.779; CI = [0.434, 0.925]) and good reliability was found in electrode sites F3, C3, C4, and P4 (0.677–0.717), with poor‐to‐fair reliability found in electrodes Pz and P3 with ICC values at 0.280 and 0.537, respectively. Agreement (SEM) was found in Tables 1 and 3.

**TABLE 3:** Alpha band (8–12 Hz).

EEG (8‐12 Hz)		Test (day 1) (24 h)	Retest (day 2) (24 h)	MD	Paired *t*‐test *p*	Effect size *d*	ICC (3,1) Consistency [CIs 95%]	SEM
		Mean ± SD (10Log(µV^2/Hz^))						
Global	Pre‐VR Exposure	−7.67 ± 4.23	−6.83 ± 5.87	−0.839	0.372	−0.297	**0.850 [0.590 0.950]**	1.983
	VR Round‐1	20.0 ± 9.16	20.5 ± 7.31	−0.444	0.873	−0.052	0.513 [−0.012 0.816]	5.571
	Rest‐1	7.72 ± 12.02	5.68 ± 9.71	2.030	0.579	0.182	0.506 [−0.021 0.813]	7.619
	VR Round‐2	17.1 ± 7.62	21.0 ± 8.75	−2.490	0.409	−0.274	**0.653 [−0.158 0.760]**	6.353
	Rest‐2	6.65 ± 8.55	7.22 ± 9.86	−0.567	0.886	−0.047	0.186 [−0.372 0.645]	8.149
	VR Round‐3	17.8 ± 7.69	18.6 ± 7.68	−0.787	0.737	−0.110	0.595 [0.107 0.852]	4.844
	Post‐VR Exposure	−7.74 ± 5.07	−4.83 ± 8.71	−2.640	0.206	−0.431	**0.630 [0.162 0.867]**	4.333
F3	Pre‐VR Exposure	−8.98 ± 4.30	−8.25 ± 4.90	−0.205	0.533	−0.205	**0.717 [0.313 0.901]**	2.441
	VR Round‐1	16.3 ± 9.84	16.6 ± 6.93	−0.312	0.916	−0.035	0.476 [−0.061 0.799]	6.074
	Rest‐1	5.13 ± 11.69	2.78 ± 8.42	2.350	0.517	0.213	0.439 [−0.106 0.782]	7.566
	VR Round‐2	16.2 ± 10.53	17.0 ± 7.59	−0.848	0.780	−0.091	0.522 [0.002 0.820]	6.271
	Rest‐2	3.68 ± 8.93	4.03 ± 9.02	−0.325	0.928	−0.093	0.188 [−0.370 0.646]	7.912
	VR Round‐3	14.3 ± 7.80	14.8 ± 6.36	−0.493	0.829	−0.070	0.551 [0.042 0.833]	4.713
	Post‐VR Exposure	−7.47 ± 5.07	−4.83 ± 8.71	−2.640	0.206	−0.431	**0.630 [0.162 0.867]**	4.333
F4	Pre‐VR Exposure	−7.51 ± 5.58	−6.46 ± 4.59	−0.414	0.727	−0.114	**0.779 [0.434 0.925]**	2.459
	VR Round‐1	19.9 ± 9.33	21.6 ± 6.83	−1.720	0.602	−0.171	0.272 [−0.291 0.695]	6.884
	Rest‐1	7.66 ± 12.04	4.56 ± 8.07	3.100	0.444	0.253	0.302 [−0.260 0.712]	8.502
	VR Round‐2	17.7 ± 7.10	21.1 ± 7.98	−3.370	0.254	−0.385	0.327 [−0.234 0.725]	6.195
	Rest‐2	6.03 ± 7.57	7.62 ± 7.34	−1.590	0.650	−0.148	0.002 [−0.519 0.523]	7.293
	VR Round‐3	15.6 ± 7.09	16.9 ± 6.96	−1.250	0.543	−0.200	**0.625 [0.154 0.865]**	4.276
	Post‐VR Exposure	−7.43 ± 5.28	−4.48 ± 9.79	−2.960	0.164	−0.479	**0.690 [0.263 0.891]**	4.377
C3	Pre‐VR Exposure	−7.86 ± 5.76	−7.40 ± 6.21	−0.466	0.773	−0.094	**0.684 [0.253 0.889]**	3.339
	VR Round‐1	21.9 ± 10.42	22.0 ± 9.41	−0.075	0.983	−0.007	0.448 [−0.096 0.786]	7.268
	Rest‐1	8.62 ± 11.1	5.27 ± 10.7	3.450	0.308	0.342	0.572 [0.072 0.842]	7.152
	VR Round‐2	18.4 ± 7.09	22.1 ± 11.01	−3.750	0.249	−0.390	0.461 [−0.079 0.792]	6.799
	Rest‐2	6.37 ± 9.72	7.92 ± 12.62	−1.540	0.772	−0.094	0.000 [−0.521 0.521]	10.990
	VR Round‐3	18.5 ± 8.90	20.3 ± 9.98	−1.760	0.515	−0.214	**0.638 [0.175 0.870]**	5.656
	Post‐VR Exposure	−5.59 ± 9.79	−6.67 ± 8.73	1.080	0.652	0.147	**0.719 [0.317 0.902]**	4.959
C4	Pre‐VR Exposure	−8.44 ± 4.71	−5.35 ± 7.70	−3.090	0.076	−0.634	**0.708 [0.296 0.898]**	3.448
	VR Round‐1	21.1 ± 8.53	17.9 ± 7.29	3.220	0.228	0.410	0.560 [−0.017 0.814]	5.560
	Rest‐1	8.55 ± 13.2	6.82 ± 11.3	1.730	0.637	0.155	**0.612 [0.133 0.859]**	7.614
	VR Round‐2	21.8 ± 8.19	20.8 ± 9.51	1.050	0.733	0.111	0.477 [−0.058 0.800]	6.337
	Rest‐2	6.69 ± 11.3	8.61 ± 11.5	−1.920	0.629	−0.158	0.465 [−0.075 0.794]	8.263
	VR Round‐3	22.3 ± 10.99	19.5 ± 8.47	2.810	0.431	0.261	0.410 [−0.142 0.767]	7.503
	Post‐VR Exposure	−7.50 ± 5.40	−4.05 ± 9.96	−3.450	0.247	−0.391	0.393 [−0.161 0.759]	6.240
P3	Pre‐VR Exposure	−5.14 ± 5.55	−7.37 ± 8.23	2.230	0.330	0.323	0.537 [−0.022 0.827]	4.773
	VR Round‐1	17.6 ± 8.68	21.1 ± 7.92	−3.520	0.266	−0.375	0.362 [−0.196 0.743]	6.633
	Rest‐1	6.37 ± 13.7	9.44 ± 12.8	−3.070	0.574	−0.185	0.242 [−0.316 0.680]	11.366
	VR Round‐2	17.2 ± 9.07	23.4 ± 10.39	−6.250	0.144	−0.505	0.197 [−0.361 0.652]	8.740
	Rest‐2	9.47 ± 10.3	8.40 ± 13.3	1.070	0.823	0.729	0.288 [−0.275 0.704]	9.859
	VR Round‐3	17.7 ± 8.63	21.7 ± 9.73	−4.020	0.198	−0.440	0.505 [−0.022 0.813]	6.471
	Post‐VR Exposure	−8.61 ± 5.22	−2.49 ± 10.48	−6.120	0.025*	−0.852	**0.623 [0.151 0.864]**	5.082
Pz	Pre‐VR Exposure	−8.75 ± 8.43	−7.14 ± 8.30	−1.610	0.633	−0.156	0.280 [−0.283 0.669]	6.994
	VR Round‐1	21.7 ± 7.79	22.3 ± 10.79	−0.607	0.852	−0.061	0.479 [−0.056 0.801]	6.697
	Rest‐1	8.39 ± 11.0	5.52 ± 11.6	2.880	0.363	0.303	**0.651 [0.196 0.875]**	6.682
	VR Round‐2	19.1 ± 5.98	21.2 ± 10.84	−2.130	0.537	−0.203	0.307 [−0.256 0.714]	7.212
	Rest‐2	7.81 ± 8.52	6.44 ± 11.02	1.270	0.746	0.106	0.296 [−0.267 0.708]	8.130
	VR Round‐3	18.5 ± 7.06	19.1 ± 9.06	−0.563	0.848	−0.063	0.427 [−0.121 0.776]	6.056
	Post‐VR Exposure	−8.25 ± 5.31	−4.82 ± 10.19	−3.420	0.223	−0.414	0.484 [−0.050 0.803]	5.836
P4	Pre‐VR Exposure	−7.65 ± 3.92	−5.86 ± 7.43	−1.790	0.266	−0.375	**0.677 [0.240 0.886]**	3.376
	VR Round‐1	21.6 ± 12.78	21.8 ± 8.53	−0.321	0.951	0.021	0.483 [−0.051 0.803]	7.698
	Rest‐1	9.29 ± 14.62	5.50 ± 8.95	3.780	0.386	0.288	0.420 [−0.130 0.772]	9.211
	VR Round‐2	19.0 ± 10.15	21.1 ± 9.73	−2.150	0.577	−0.183	0.332 [−0.229 0.728]	8.026
	Rest‐2	6.60 ± 8.79	7.51 ± 8.98	−0.909	0.832	−0.069	0.000 [−0.521 0.521]	8.661
	VR Round‐3	17.4 ± 7.90	17.6 ± 8.76	−0.233	0.911	−0.036	**0.728 [0.334 0.906]**	4.317
	Post‐VR Exposure	−7.46 ± 4.81	−4.95 ± 7.78	−2.510	0.173	−0.469	**0.657 [0.206 0.878]**	3.792

*Note*: Reliability (intraclass correlation coefficient; ICC), agreement (standard error of measurement; SEM), and mean difference (MD) for the average global (i.e., across all electrodes) and local (i.e., at each electrode site; F3, F4, C3, C4, P3, Pz, and P4) across the alpha band (8–12 Hz). EEG metrics were recorded for pre‐virtual reality (VR) exposure during three rounds of VR (denoted as VR Round‐1, VR Round‐2, and VR Round‐3, respectively) during two intermediate rest intervals between each VR round (denoted as Rest‐1 and Rest‐2, respectively), and during a post‐VR exposure condition. CIs = 95% confidence intervals; SD = standard deviation; *n* = 10. ICC ≥ 0.6 (i.e., good and excellent) are represented in bold.

*p < 0.05.

Similarly, neither main effects were reported on the global EEG results for the post‐VR exposure EEG (*F*(1,10) = 1.860, *p* = 0.106, *η* ^2^ = 0.037), nor for any electrode placements (*p* > 0.05; see Table 1), apart from electrode P3 (*p* = 0.025). Global EEG metrics showed good test‐retest reliability (0.630; CI = [0.162, 0.867] for the post‐VR exposure EEG. When investigating individual electrode sites, excellent reliability was displayed in electrode placement C3 (0.719; CI = [0.317, 0.902]) and good reliability was found in electrode sites F3, F4, P3, and P4 (0.623–0.690), with poor‐to‐fair reliability found in electrodes C4 and Pz with values of 0.393 and 0.484, respectively. Agreement (SEM) was found in Tables 1 and 3.

#### 3.3.3 Average VR EEG

No main effects of the sessions on the global EEG results were reported for either task‐active VR intervals (*p* = 0.907, 0.344, and 0.742, for VR Round‐1, VR Round‐2, and VR Round‐3, respectively), as well as for all electrode placements (*p* > 0.05). Global EEG metrics over the specified frequency bands showed poor‐to‐fair reliability (0.393–0.591) across the task‐active VR intervals. When investigating individual electrode sites, good reliability was displayed in electrode placements F4, C3, and P4 (0.634–0.697; reported in VR Round‐3), with other electrode placements displaying poor‐to‐fair reliability (0.262–0.697) across the VR intervals within the test‐retest sessions. Agreement (SEM) was found in Tables 1 and 2.

#### 3.3.4 Alpha VR EEG

No main effects of the sessions on the global EEG results were reported for either task‐active VR intervals (*p* = 0.873, 0.409, and 0.737 for VR Round‐1, VR Round‐2, and VR Round‐3, respectively), as well as for all electrode placements (*p* > 0.05). Global EEG metrics over the specified frequency bands showed fair‐to‐good reliability (0.513–0.636) across the task‐active VR intervals. When investigating individual electrode sites, reliability ranged from poor (e.g., electrode P3 in VR Round‐2; ICC = 0.197 [‐0.361 0.680]) to excellent (e.g., electrode P4 in VR Round‐3; ICC = 0.728 [0.206 878]) across the VR intervals within the test‐retest sessions. Agreement (SEM) was found in Tables 1 and 3.

#### 3.3.5 Average Rest Interval EEG

No main effects were reported from the sessions on the global EEG results. This was found neither for the first rest interval (Rest‐1; F(1,10) = 0.157, *p* = 0.702, *η* ^2^ = 0.005), the second rest interval (Rest‐2; F(1,10) = 0.078, *p* = 0.786, *η* ^2^ = 0.004), nor for any individual electrode placements (*p* > 0.05). Averaged global EEG metrics showed fair (Rest‐1 = 0.506 [−0.020 0.759]) and poor (Rest‐2 = 0.208 [−0.352 0.658]) test‐retest reliability for the rest intervals in order of sequence. Variability was found within individual electrode sites, with reliability ranging from poor to good (0.000–0.674). Agreement (SEM) was found in Tables 1 and 2.

#### 3.3.6 Alpha Rest Interval EEG

No main effects were reported from the sessions on the global EEG results. This was found neither for the first rest interval (Rest‐1; F(1,10) = 0.332, *p* = 0.579, *η* ^2^ = 0.009), the second rest interval (Rest‐2; F(1,10) = 0.022, *p* = 0.886, *η* ^2^ = 0.001), nor for any individual electrode placements (*p* > 0.05). Averaged global EEG metrics showed fair (Rest‐1 = 0.506) and poor (Rest‐2 = 0.208) test‐retest reliability for the rest intervals in order of sequence. Variability was found within individual electrode sites, with reliability ranging from poor to good (0.000–0.674). Agreement (SEM) was found in Tables 1 and 3.

#### 3.3.7 Agreement

**FIGURE 5:**
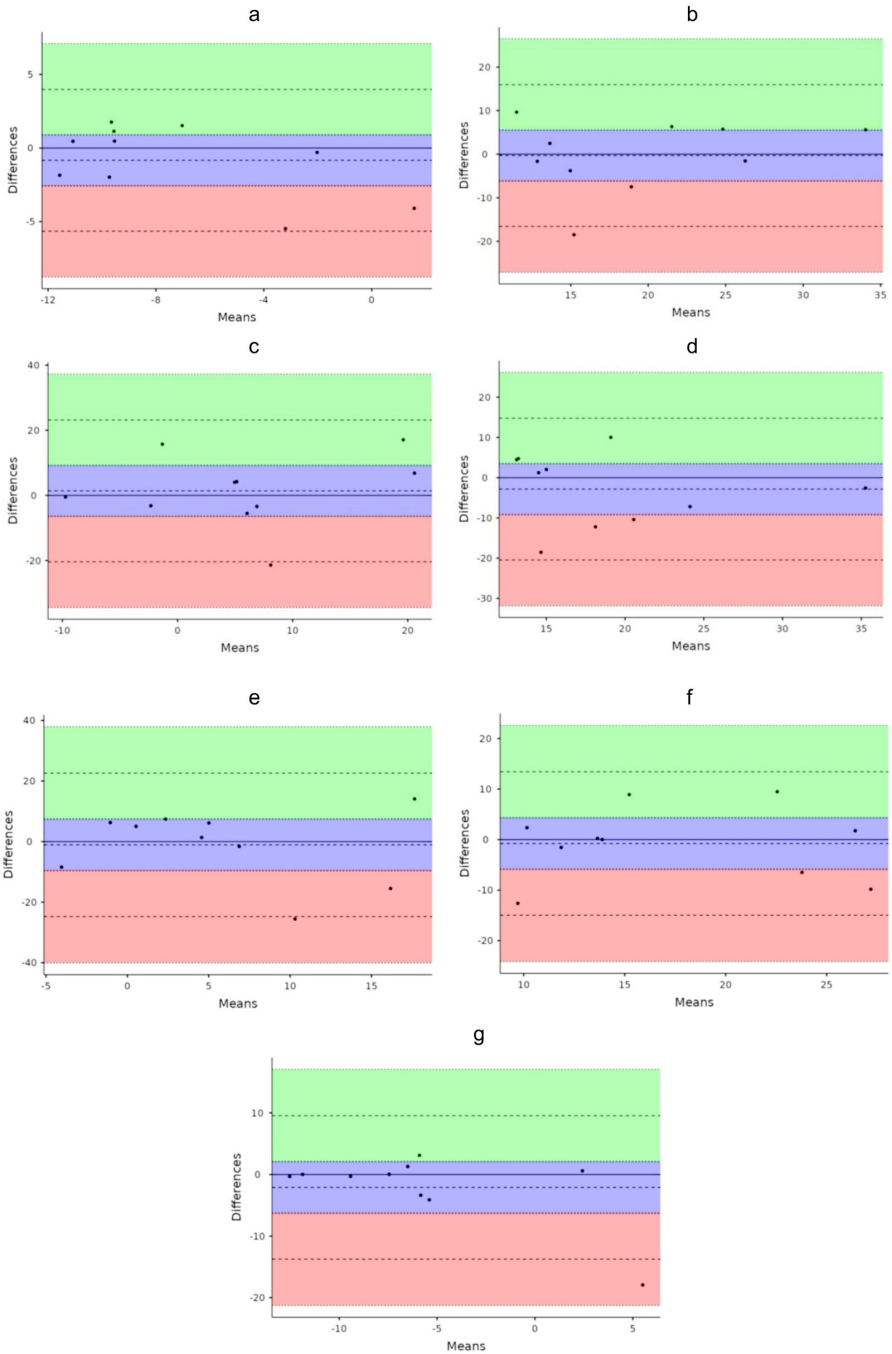
Bland–Altman plots for the continuous EEG data, comparing the test and retest sessions. The plots display the global 10log_10_ transformed power spectrum density (PSD) differences between the sessions, against each participant's mean. These are presented for the (a) pre‐virtual reality (VR) exposure, (b) VR Round‐1, (c) the first rest interval (Rest‐1), (d) VR Round‐2, (e) the second rest interval (Rest‐2), (f) VR Round‐3, and (g) post‐VR exposure.

Agreement measures using the typical error (SEM) showed that differences between the test‐retest measurements were small, with values starting from 1.717 upwards (see Tables 1, 2, 3). Across the protocol, errors of measurement ranged between 4.000 a d 6.000 across all frequency bands. However, higher SEM values were consistently noted within the Rest‐1 and Rest‐2, with values ranging from 7.147 to 11.108 across measurement parameters. Bland–Altman plots (Figure 5) displayed mean difference bias is close to zero, indicating the mean differences between test‐retest sessions were in agreement across the protocol. Consistent differences across measurements magnitudes were found, with no evident proportional biases and/or heteroscedasticity.

## 4 Discussion

The present study examined the recording feasibility and test‐retest (24 h) reliability of 4‐ to 30‐Hz EEG bands using a dry‐electrode EEG system during a dynamic psychomotor VR task. The main findings of this study were that (1) the EEG system offered feasibility in the extraction of relevant neurophysiological information, in the form of the PSD values of interest during the VRex (i.e., motion), and (2) overall the dry‐electrode EEG displayed reliability ranging from poor to excellent (0.208–0.858) across measurements. Such findings were globally consistent, both across all spectral bands independently (i.e., theta, alpha, low beta, and high beta), as well as averaged across the specified frequency range.

### 4.1 Standardization Evaluation

The results confirmed no significant differences in HR, relative HR, punch count, punch speed or perceived effort between the Test and Retest sessions. Small effect size for all variables, other than perceived exertion (small‐to‐medium effect size), further suggests the VR‐boxing protocol conditions between test and retest were matched for intensity. Such results demonstrate the standardization of the protocol.

In comparison with previous research reporting on HR during simulated boxing (Finlay et al. 2020; Thomson and Lamb 2017), it was identified that HR and relative HR % in this study also increased with consecutive rounds during each testing session. The relative HR intensities were also matched between test and retest days but in comparison to other studies the average relative intensities were ∼10% lower than those quoted for amateur boxers in simulated boxing experiments (Finlay et al. 2020; Thomson and Lamb 2017). One explanation may be the variation in striking style, and thus range of relative physiological load, between different combat‐sports participants in this study. An additional explanation may be the constrained combos to standardize the VRex (i.e., hooks and uppercut were not allowed) requiring a lower effort overall.

The average punch velocity values recorded in this study are comparable to other literature that reported hand‐accelerometer velocities (Stanley et al. 2018) but slightly lower velocities to similar punch trackers as used in this study for jab and cross punching styles (Omcirk et al. 2023). It could be explained by the lack of the competitive element, compared to other studies, may have impacted on the willingness to maximize punch speed.

### 4.2 EEG Recording Feasibility—Motor‐Artifacts

The present data showed that the EEG system can detect meaningful signal during VR‐induced motion, indicating feasibility in the extraction of potentially relevant neurophysiological information, in the form of the PSD values of interest. ICA was employed as standardized method of consistently and uniformly dealing with unwanted artifacts. Such method involved the identifying and removing of unwanted components which surpassed a specified threshold of artifact characterization (Delorme and Makeig 2004). As detected by EEG electrodes, motion artifacts are large interfering signals induced by the users’ motion (Beach et al. 2021). Such artifacts may be either due to electrode displacement against the scalp or changes on the surface of the skin. For instance, if proper contact is not made by the electrodes on the scalp, motion artifacts can be induced, thereby corrupting EEG signals. This is of particular concern when using EEG in high‐motion and dynamic sports (C. H. Wang et al. 2019). Relevantly, dry‐electrode EEG technology is designed to minimize relative motion between the electrodes and the user, by means of maintaining constant contact on the scalp (Seok et al. 2021; Taheri et al. 1994). For this reason, such technology offers the potential of EEG collection during sports (Kohli and Casson 2015). As the DSI‐7 has previously shown feasibility in exacting PSD values during sporting conditions (Kohli and Casson 2015), the present results further support the use of the dry‐electrode technology during dynamic movements/tasks, such as those induced by VRex‐based training.

### 4.3 Global EEG Reliability

#### 4.3.1 VRex

Within the task‐active VR intervals, global EEG metrics within the alpha band showed fair‐to‐good reliability (0.513–0.636), although showed poor‐to‐fair reliability (0.393–0.591) when using the averages across the specified frequency range. Agreement was evaluated in the form of typical error (SEM) and was found to be in a consistently acceptable range. Bland–Altman plots confirmed absolute agreement between test‐retest sessions across the protocol. The alpha band results thereby indicate the potential employment of such EEG metrics for the investigation of neural mechanisms associated with dynamic VRex task, although further measures to increase the reliability may be required (as discussed in sections to follow).

Overall, although variability in test‐retest reliably is found within frequency bands (Höller, Uhl, et al., 2017), the use of power of frequency band investigation in adults displays excellent (0.76–0.99) reliability across most bands and relational band measures (Lopez et al. 2023). For instance, it has been suggested that the averaging of measures within specified frequency ranges is suggested to increase reliability with EEG‐based metrics, and thereby leading to more reliable results (e.g., in the form of Spearman rank correlation; Höller, Uhl, et al., 2017). For this reason, the present study employed averaged power spectrum analysis over the specified frequency bands as one means to investigate reliability. The alpha band was investigated independently, in alignment to the primary investigation (i.e., centering the alpha band). As confirmed by the present results, the frequency band of investigation did indeed impact the reliability of EEG within the VRex bout.

Interestingly, lower reliability has been demonstrated within power values of lower frequencies, such as those within the delta range compared to higher frequency bands (Hatz, Hardmeier, Bousleiman, et al. 2015). Further, lower reliability is found in theta and beta frequency band, whereas the alpha band displays higher reliability as well as higher variability (Hardmeier et al. 2014; Höller, Uhl, et al., 2017). The present results showing higher reliability within the alpha compared to averages across a 4‐ to 30‐Hz range are thereby in alignment with work displaying the highest reliability being found centering the alpha band compared to both lower (i.e., delta) and higher (i.e., gamma) frequency ranges (Hatz, Hardmeier, Bousleiman, et al. 2015; Höller, Uhl, et al., 2017).

#### 4.3.2 Pre‐ and Post‐VR Exposure Rest EEG

The inter‐day tests revealed the reliability ranging from poor to excellent, across the pre‐ and post‐VR exposure EEG, when investigating the alpha and the average (4–10 Hz) frequency range. Such resting EEG measurements provide a baseline reliability for metrics with the dry‐electrode EEG system in a non‐fatigued condition (i.e., before a bout of VRex training), and a resting EEG measurement after enduring the neurophysiological demands of the VRex training. For the pre‐VR exposure EEG, global EEG metrics showed excellent test‐retest reliability, with ICC values ranging from 0.850 to 0.858 for the alpha and average frequency range, respectively. These results were in alignment with other studies showing excellent test‐retest reliability of continuous EEG in the power analysis at rest (Corsi‐Cabrera et al. 2007; Gudmundsson et al. 2007; McEvoy et al. 2000; Williams et al. 2005) and were expected due to the systems’ requirement to comply technological standards. As for the post‐VR exposure, resting EEG displayed reliability ranging from fair (0.575 for the average band) to good (0.630 for the alpha band). As such findings are assumed to capture neuropsychological changes associated with the bout of VRex training, the decrease in reliability within the post condition is speculated to be due to sweat and/or movement of electrodes (i.e., causing reliability to deteriorate relative to the pre‐VR exposure baseline), or the buildup of fatigue (Zhang et al. 2022).

In general, excellent reliability has been found using frequency band investigation during baseline conditions, although it must be noted that these results were found over time periods longer than a year (Lopez et al. 2023). Over long‐time ranges, the absolute power of traditional frequency bands shows high variance between subject and notably high test‐retest reliability within subjects (Dustman et al. 1999; Gasser et al. 1985; Grandy et al. 2013; Näpflin et al. 2007). Another study investigating test‐retest reliability held over longer durations revealed ICC ranging from 0.68–0.80 and 0.12–0.73 for global interactions and graph measures, respectively (Hardmeier et al. 2014). Regarding shorter terms (i.e., weeks), test‐retest protocols are found to range in from low reliability (i.e., below 0.5) to high reliability (i.e., above 0.9; Höller, Butz, et al. 2017). As the present study used short durations of investigation (i.e., over 24 hours), it is suggested that the timescale of investigation may have contributed to the variation found within the results.

#### 4.3.3 Rest Intervals

The global EEG metrics showed poor to fair (0.208–0.506) test‐retest reliability for the rest intervals across the average and alpha frequency range. Such results indicate that the dynamics of VRex protocol impacted the reliability of resting EEG conditions, which interestingly was also found when comparing the pre‐ and post‐VR exposure resting EEG. For instance, such impacting variables may involve changes in precision and accuracy requirements of the VR boxing exercise, inducing moderation in attention moderation, reaction time, and focus (Wu et al. 2024; Yordanova et al. 2023). Therefore, a possible interpretation of such result may be reflecting neuropsychological changes associated with, for example, cognitive fatigue due to the bout of VRex training (Zhang et al. 2022). In addition, the low reliability found in these intermediate rest intervals may additionally to be a result of factors impacting contact endured as a result of the VRex bouts (i.e., sweat, movement of electrodes; Kalevo et al. 2020; Kohli and Casson 2015; Tatum et al. 2011). For this reason, it is suggested that further measures between dynamic bouts of EEG measurement (such as additional electrode adjustment, cleaning) may be required in order to maintain the integrity of the detected signal.

### 4.4 Local EEG Reliability

Within the pre‐ and post‐VR exposure EEG conditions, electrode sites ranged from poor to excellent in both the average and alpha frequency range. As the post‐VR exposure EEG electrode reliability ranged from poor to good in the average PSD range (compared to poor to excellent in the pre exposure condition), such results indicate that the reliability of the system was impacted by the VRex protocol. Interestingly, the alpha band seemed to be less impacted by the protocol, wherein this band showed greater reliability over the average frequency range across electrode sites. This is consistent with findings showing the highest ICC reliability being found centering the alpha band for baseline measures compared to surround bands (e.g., beta or theta bands; Hardmeier et al. 2014; Höller, Uhl, et al., 2017). The decrease in reliability on a local scale is additionally assumed to derive from movement of the electrodes endured during the VRex task, as well as other factors associating with endured effort, such as sweat (Kalevo et al. 2020; Kohli and Casson 2015; Tatum et al. 2011), although the ergonomics of the headset used in the present work may also account for the variation found in local reliability.

Importantly, investigating such assumption on a local scale (i.e., per each electrode) allows for insight on whether certain sites are more susceptible and/or prone to be impacted by moderating variables (such as movement as a result of the protocol). For instance, within the present work, electrodes F3 and C3 presented as the electrodes impacted by the VR protocol (decreasing from 0.707 to 0.417 and from 0.685 to 0.396, respectively), from the pre‐ to post‐VR resting EEG measurements. This indicates that under the parameters of the present study, such electrodes either 1) require increased interventional care throughout the protocol beyond what was already implemented, 2) may not serve as reliable measurement sites for this VRex‐based protocol, or 3) may have been affected by suboptimal headset ergonomics for the dynamic task. Further investigation is required to support these claims.

In extension, local investigation may provide insight on which electrodes may be most suitable for investigations involving dynamic VR training. Although no electrodes consistently displayed excellent reliability across all measures, electrode placements C3 and F4 were identified to be most consistently reliable. Such results indicate that electrode placements therefore potentially act as reliable sites of EEG measurement during VRex‐based investigations. As such, it is important to note that factors impacting electrode reliability have been previously explored and therefore may have impacted the present findings. For one, age has been shown to impact the reliability of EEG measures with older adults showing higher reliability at the fronto‐central site (Cz) with young adults having higher reliability at the centro‐parietal area site (Pz) within the same EEG measure (e.g., P3 amplitude; Walhovd and Fjell 2002). Further, variability in reliability associated with age has been found within powers of frequency bands or over specific scalp topography in adults (i.e., ranging from fair to excellent; Lopez et al. 2023). Alternatively, over the scale of weeks, excellent test‐retest reliability is found within younger (Angelidis et al. 2016) and older adults (Keune et al. 2019). For this reason, it is necessary to consider that the variation of the results of the present study may have been impacted by the reliability of electrode sites associated with factors, such as age. However, further evidence to support such claim is required.

### 4.5 General Reliability of EEG Measures

Given the varying reliability observed across measurements in the present study, it is important to acknowledge that the issue of variable reliability in individual differences is widely recognized in neuroscience (Elliott et al. 2021; Greene et al. 2022; Kennedy et al. 2022; Kragel et al. 2021; Lopez et al. 2023; Noble et al. 2021). This challenge has been particularly emphasized in the context of individual differences analysis in methodologies such as EEG and magnetic resonance imaging (MRI; Lopez et al. 2023), leading to the characterization of cognitive neuroscience as being “at a crossroads” (“Cognitive Neuroscience at the Crossroads” 2022). Generally, the test‐retest reliability of EEG has been shown to be highly variant and ultimately depends on various implications and factors. These include frequency, trial number, signal‐to‐noise ratio (Miskovic and Keil 2015), epoch length (Gudmundsson et al. 2007), recording intervals (Sandman and Patterson 2000), reference schemes (Towers and Allen 2009), as well as different metrics of the EEG data (Tenke et al. 2018). For instance, lower reliability is found in phase‐dependent measures compared to non‐phase‐dependent metrics, such as power and coherence (Cannon et al., 2012). Relevantly, the frequency band of investigation plays an important role in the reliability of the investigation at hand, where the form of measure can be said to account for differences between reliability within frequency ranges (Höller, Uhl, et al., 2017). For this reason, the present study used power frequency band investigation as this has been shown to be highly reliable across most bands and relational band measures in adults (Lopez et al. 2023).

It is important to mention that the similarities found in test‐retest reliability (e.g., as measured by intraclass correlation coefficient; Bartko 1966; Fisher 1958; Yen and Lo 2002) derive from the characteristics and estimates of individual's data obtained within a session, which are highlighted to be accounted for by psychometric reliability. Psychometric reliability characterizes internal consistencies and self‐similarities in repeated measures (as defined by: Cronbach 1951; Streiner 2003; Strube et al. 2007), where research investigating individual differences in brain metrics is highly constrained by (Lopez et al. 2023; Parsons et al. 2019). Such mention thereby encapsulates the variability found in test‐retest reliability, although other factors such as accompanying brain network also play a role in the variability found in EEG reliability (Höller, Uhl, et al. 2017). Ultimately, the variability in EEG data is believed to derive from the non‐stationary nature of the EEG (Hatz, Hardmeier, Benz, et al. 2015), although further investigation is needed to explain the variability in the reliability of these neurophysiological metrics (Höller, Uhl, et al. 2017).

### 4.6 Limitations, Applications, and Future Work

As supported by the present work, the combination of VR and EEG holds potential for understanding the mechanisms behind athlete performance, investigating training regimens, and advancing our understanding of the interplay between the mind and body in sports (Fahl et al. 2023; Richlan et al. 2023; J. Wang 2012). For instance, head‐mounted VR technology has been shown to support the delivery of realistic first‐person perspective embodied feedback, in turn, increasing intra‐individual awareness, thereby improving motor learning (Haar et al. 2021; Kilteni et al. 2012). In combination with the EEG systems’ ability to support real‐time assessment of brain activity patterns, the neural adaptations during VR‐based training need be monitored, examined, and thereby utilized. This exists in the form of real‐time neurofeedback training, which has been shown to enhance performance by providing immediate feedback on brain activity patterns (Berger and Davelaar 2018; Gong et al. 2021; Keune et al. 2019; J. Morrone and Minini 2023). Beyond sports, VR also holds promise in rehabilitation by enhancing recovery in stroke, brain injury, and other neuromuscular disorders through neuroplasticity and adaptive training (Ceradini et al. 2024; Khan et al. 2023; Perez‐Marcos 2018). When paired with EEG‐driven feedback, real‐time neurophysiological monitoring during immersive, motor‐based interventions, may allow for personalized rehabilitation and optimized outcomes through brain‐informed strategies (Z. Wang et al. 2022).

Additionally, potential longitudinal monitoring (i.e., across multiple sessions) may be supported for the tracking of EEG‐based neurophysiological indices associated with task‐specific performance over time (Morrone and Pedlar 2024a; Morrone and Minini 2023). In the next steps to achieve this, the investigation and characterization of the manner in which both the periodic and aperiodic EEG signal alter as a result of the dynamic training, especially in relation to the individual differences, should be done (Gyurkovics et al. 2021). As such, the present study highlights the potential to employ the combination of VR and EEG, enabling researchers and coaches to utilize EEG markers, such as alpha band activity, for monitoring attention in sport, potentially facilitating performance optimization and cognitive training.

However, monitoring EEG during sports poses challenges, whereby traditional wired EEG systems limit natural and dynamic motion, are highly susceptible to motion artifacts, may face electrode placement issues, and involve highly specific operational requirements which require a specialist to conduct (Zander et al. 2011). Dry‐electrode technology, such as that used in the present work, offers a means to overcome such challenges. Although this might be the case, it must be highlighted that such systems may involve user discomfort, endure environmental interference, and the complexity of data interpretation remains difficult (C. H. Wang et al. 2019). Additionally, as dry‐electrode systems remain susceptible to motion artifacts, further work investigating suitable artifact characterization methods associated with highly dynamic and/or psychomotor‐based tasks is recommended in order to support the implementation of such tools within practical applications and real‐world settings. This may be supported in the form of targeted signal processing techniques, such as artifact removal algorithms or adaptive filtering to minimize the impact of motion artifacts on EEG data (Beach et al. 2021).

It should also be noted that although the present system was more fitted for dynamic investigations than traditional EEG systems, it remained somewhat cumbersome as well as had limited number of electrodes, therefore restricting the informativeness of the topographical maps (e.g., of posterior cortical activity). In addition, the study would have benefitted from a larger sample size. Value would also be found in conducting associated validation studies to assess the performance of such dry‐electrode EEG systems in other alternative high‐motion movements and environments (i.e., other VRex simulations), and/or work monitoring or manipulating of head movements to either control or compensate for the motion artifacts induced by such movements. It may also be suggested to compare such EEG technology during sports activities with data collected during more controlled, low‐motion conditions to evaluate related changes in signal quality and reliability. With this stated, it is argued that the present results provide valuable insight into the potential neuronal alterations endured as a result VRex‐based training, although further investigation on the basis of these results is warranted.

## 5 Conclusion

The present study examined the test‐retest reliability of a dry‐electrode EEG system during a dynamic psychomotor VR task, over 2 consecutive days. Inter‐day analysis was used to investigate the EEG signal pre, during, post and a 3‐ by 3‐min VRex boxing focus ball protocol. Due to dry‐electrode EEG headsets designed to comply with the needs of clinical applications, we expected to find moderate‐to‐excellent reliability of the metrics analyzed. The EEG system offered feasibility in the extraction of the metrics of interest, which was achieved under confirmed standardized parameters.

The dry‐electrode EEG displayed reliability ranging from poor to excellent (0.208–0.858) across measurements. Such findings were globally consistent both across all spectral bands independently (i.e., theta, alpha, low beta, and high beta), as well as averaged across the specified frequency range. For this reason, the findings indicate the potential viability of using dry‐electrode EEG technology for the investigation of neural mechanisms associated with such protocol. Such results provide preliminary data on the reliability of using neuroelectric activity, such as the alpha and surrounding frequency bands, for assessing the cognitive demands associated with psychomotor‐based VR tasks.

## Author Contributions

**J. Morrone**: conceptualization; data collection; EEG analysis; methodology; writing‐original draft, writing‐review and editing. **R. Mellor**: conceptualization, data collection; heart rate, perception of effort and punch output analyses; writing‐original draft, writing‐review and editing. **S. Grosprêtre**: conceptualization; methodology; writing‐review and editing; **C. Pedlar**: conceptualization; writing‐review; supervision. **G. Cimadoro**: conceptualization; methodology; heart rate, perception of effort and punch output analyses, writing‐original draft; writing‐review and editing, supervision.

## Ethics Statement

Ethical approval was granted by the St Mary's University Ethics Committee (SMU_ETHICS_2023‐24_494) in accordance with the ethical standards established in the Declaration of Helsinki. Informed written consent was provided by all participants.

## Conflicts of Interest

The authors declare no conflicts of interest.

### Peer Review

The peer review history for this article is available at https://publons.com/publon/10.1002/brb3.70448.

## Data Availability

The data that support the findings of this study are openly available from Mendeley Data at https://doi.org/10.17632/c3sw32zn4m.1.

## Appendix 1

### EEG Data Pre‐Processing

A uniform pre‐process standardized procedure was initially undergone, which involved data segmentation and the consecutive processing of independent component analysis (ICA). Such procedure involved the identifying and removing of unwanted components, achieved through both filters and visual inspection‐based artifact rejection analysis (Delorme and Makeig 2004). Components that were rejected were those predicated to account for non‐brain activity at a magnitude of equal to or above 90%, including oculomotor artifact and visual contextual complexes (such as eye‐blinks and eye‐movements), muscular activities, and other non‐brain related complexes (Jung et al. 2000). As a result, out of all EEG files a total of 117 (<12%) independent components (IC) were removed.

The power spectrum densities (PSD) were quantified through fast Fourier transform using Welch's method with windows of 500 sampling points and 375 sampling point overlaps. As the present study predominantly targets activity surrounding the alpha band, only the theta, alpha, and beta bands were evaluated. The PSDs were extracted per participant per protocol and were decomposed into the following frequency bands: theta (4–7 Hz), alpha (8–12 Hz), low beta (13–16 Hz), and high beta (17–30 Hz). The beta band was broken into its lower and higher constituents to evaluate if the components of band closer the alpha band (i.e., low beta) offered variation in reliability compared to its higher frequency counterparts. PSD values were then 10log_10_ transformed to achieve higher degrees of normal distribution; therefore, interpretations of individual frequency bands should be considered on respective scales.
